# Queuine Is a Nutritional Regulator of Entamoeba histolytica Response to Oxidative Stress and a Virulence Attenuator

**DOI:** 10.1128/mBio.03549-20

**Published:** 2021-03-09

**Authors:** Shruti Nagaraja, Maggi W. Cai, Jingjing Sun, Hugo Varet, Lotem Sarid, Meirav Trebicz-Geffen, Yana Shaulov, Mohit Mazumdar, Rachel Legendre, Jean-Yves Coppée, Thomas J. Begley, Peter C. Dedon, Samudrala Gourinath, Nancy Guillen, Yumiko Saito-Nakano, Chikako Shimokawa, Hajime Hisaeda, Serge Ankri

**Affiliations:** a Department of Molecular Microbiology, Ruth and Bruce Rappaport Faculty of Medicine, Technion, Haifa, Israel; b Department of Microbiology and Immunology, National University of Singapore, Singapore; c Singapore-MIT Alliance for Research and Technology, Singapore; d Department of Biological Engineering, Massachusetts Institute of Technology, Cambridge, Massachusetts, USA; e Plate-forme Technologique Biomics, Centre de Ressources et Recherches Technologiques (C2RT), Institut Pasteur, Paris, France; f Hub de Bioinformatique et Biostatistique, Département Biologie Computationnelle, USR 3756 CNRS, Institut Pasteur, Paris, France; g Jawaharlal Nehru University School of Life Sciences, New Delhi, India; h Department of Biological Sciences, The RNA Institute at the University at Albany, State University of New York at Albany, Albany, New York, USA; i Centre National de la Recherche Scientifique, CNRS-ERL9195, Paris, France; j Institut Pasteur, Paris, France; k Department of Parasitology, National Institute of Infectious Diseases, Shinjuku-ku, Tokyo, Japan; University of California Los Angeles

**Keywords:** *Entamoeba histolytica*, oxidative stress, tRNA modification, virulence

## Abstract

Queuosine is a naturally occurring modified ribonucleoside found in the first position of the anticodon of the transfer RNAs for Asp, Asn, His, and Tyr. Eukaryotes lack pathways to synthesize queuine, the nucleobase precursor to queuosine, and must obtain it from diet or gut microbiota. Here, we describe the effects of queuine on the physiology of the eukaryotic parasite Entamoeba histolytica, the causative agent of amebic dysentery. Queuine is efficiently incorporated into E. histolytica tRNAs by a tRNA-guanine transglycosylase (EhTGT) and this incorporation stimulates the methylation of C38 in ${\text{tRNA}}_{\text{GUC}}^{\text{Asp}}$. Queuine protects the parasite against oxidative stress (OS) and antagonizes the negative effect that oxidation has on translation by inducing the expression of genes involved in the OS response, such as heat shock protein 70 (Hsp70), antioxidant enzymes, and enzymes involved in DNA repair. On the other hand, queuine impairs E. histolytica virulence by downregulating the expression of genes previously associated with virulence, including cysteine proteases, cytoskeletal proteins, and small GTPases. Silencing of EhTGT prevents incorporation of queuine into tRNAs and strongly impairs methylation of C38 in ${\text{tRNA}}_{\text{GUC}}^{\text{Asp}}$, parasite growth, resistance to OS, and cytopathic activity. Overall, our data reveal that queuine plays a dual role in promoting OS resistance and reducing parasite virulence.

## Abstract (precis)

Entamoeba histolytica is a unicellular parasite that causes amebiasis. The parasite resides in the colon and feeds on the colonic microbiota.

## INTRODUCTION

Amebiasis is an enormous global medical problem due to poor sanitary conditions and unsafe hygiene practices in many parts of the world. According to the World Health Organization, 50 million people in India, Southeast Asia, Africa, and Latin America suffer from amebic dysentery, with amebiasis causing the death of at least 100,000 people each year. Entamoeba histolytica, the etiologic agent of amebiasis, proliferates in the intestinal lumen and phagocytoses resident gut flora. Over the last few decades, it has become evident that E. histolytica’s pathogenicity is directly linked to the parasite’s interaction with the gut microbiota (1). This interaction is very selective because only those bacteria with the appropriate recognition molecules are ingested by the parasite (2). It has been reported that E. histolytica’s association with specific intestinal bacteria changes the parasite’s cell surface architecture (3, 4). It has also been reported that phagocytosis of pathogenic bacteria boosts E. histolytica’s cytopathogenicity, increases the expression of Gal/GalNAc lectin on the cell surface, and boosts cysteine proteinase activity when trophozoites are cocultured with the enteropathogenic Escherichia coli (EPEC) O55 or Shigella dysenteriae (5). It has also been reported that bacterium-induced augmentation of E. histolytica’s virulence is achieved only when the trophozoites phagocytose intact live cells (6). The composition of the gut flora in patients suffering from amebiasis shows a significant decrease in the population sizes of *Bacteroides*, Clostridium coccoides, Clostridium leptum, *Lactobacillus*, and *Campylobacter* and an increase in *Bifidobacterium*, while there was no change in *Ruminococcus* compared to that in healthy patients (7). These findings suggest that the pathogenesis of amebiasis might be driven by a dysregulated microbiome or cross talk between enteropathic bacteria, the parasite, and the intestinal immune system. This cross talk may be modulated by chemical signaling molecules, such as short-chain fatty acids (SCFAs) released by the bacteria (8), or by bacterial oxaloacetate that regulates parasite virulence and resistance to oxidative stress (OS) (9, 10). The modified ribonucleoside, queuosine, or 7-{[(4.5-*cis*-dihydroxy-2-cyclopenten-1-yl)-amino]-methyl}-7-deazaguanosine, may also participate in the cross talk between the parasite and the microbiota. *Eubacteria* can synthesize the queuine nucleobase precursor of queuosine *de novo*. However, a recent study showed that some *Eubacteria* can also salvage precursors of queuosine (11). Since mammals cannot synthesize queuine and salvage it from diet and gut microbes, these observations suggest a dynamic supply and demand for queuine by both the gut microbiota and the host (12–14). The diverse roles of queuosine in bacterial physiology are only now emerging. tRNA-guanine transglycosylase (TGT), which catalyzes the exchange of queuine for guanine at the wobble position of certain tRNAs, has been recognized as one of the key enzymes that regulate virulence in Shigella flexneri (15, 16). Recently, it has been found that a modification of the wobble position of tRNA with a GUN anticodon by 7-deaza-guanosine derivative queuosine (Q34) stimulates Schizosaccharomyces pombe Dnmt2/Pmt1-dependent C38 methylation (m^5^C38) in the ${\text{tRNA}}_{\text{GUC}}^{\text{Asp}}$ anticodon loop (17), with the latter correlated with resistance to OS in many organisms, including E. histolytica (18, 19). The fact that both methylation of C38- ${\text{tRNA}}_{\text{GUC}}^{\text{Asp}}$ and queuinosylation of tRNAs occur at the same anticodon loop suggests coordination among these modifications in regulating OS resistance in the parasite. It was previously reported that queuine promotes the resistance of cancer cells to OS by increasing the activity of antioxidant enzymes (20). In contrast, queuosine deficiency in tRNAs leads to the accumulation of misfolded proteins that induce a cellular stress response (17). It has also been found that queuine enhances translational speed and fidelity in eukaryotes (21).

Here, we report that queuine (i) induces C38 hypermethylation (m^5^C38) in the ${\text{tRNA}}_{\text{GUC}}^{\text{Asp}}$ anticodon loop, (ii) promotes resistance of the parasite to OS by triggering the expression of genes associated with stress response, (iii) restores protein synthesis in the parasite exposed to OS, and (iv) reduces parasite virulence by reducing the expression of genes associated with virulence.

(This article was submitted to an online preprint archive [22].)

## RESULTS

### Queuine is incorporated into E. histolytica tRNA and induces C38 hypermethylation (m^5^C38) in the ${\text{tRNA}}_{\text{GUC}}^{\text{Asp}}$ anticodon loop.

Given the established role of queuine as a precursor to queuosine (Q) in tRNA, we first set out to characterize the incorporation of queuine into amoebic tRNA. Here, we quantified Q in tRNA in the parasite using liquid chromatography-tandem mass spectrometry (LC-MS/MS). tRNA samples were prepared from untreated trophozoites and from trophozoites that were grown with queuine (0.1 μM). This concentration of queuine was chosen because (i) it provides to the parasite the best protection against H_2_O_2_ (see Fig. S1 in the supplemental material available at https://datadryad.org/stash/share/Y6qAAIhEzUcPNlK16zSmOvPJyjd0bqUcPUz3mNw1fCg) and (ii) because it has also been used in a study on the social amoeba Dictyostelium discoideum (23). The amount of queuine in the human large intestine is unknown. However, its amount in the blood can reach 10 nM (24). A 10- to 1,000-fold level of difference can be normally observed between the level of a metabolite in the large intestine and its level in the blood (25). Therefore, the concentration of queuine used in this study (0.1 μM) is probably biologically relevant.

**FIG 1:**
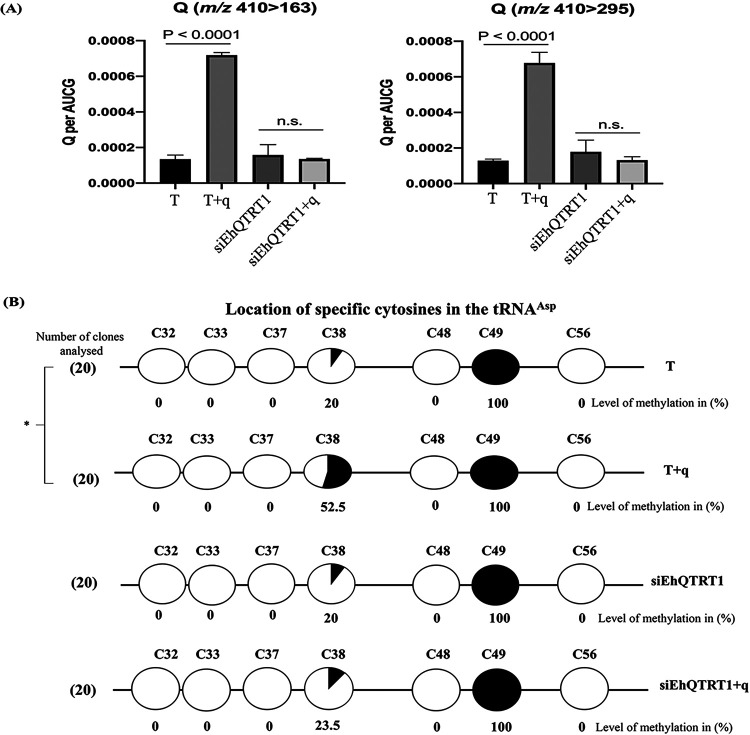
Queuine is efficiently incorporated into E. histolytica tRNA and leads to C38 hypermethylation (m^5^C38) in the anticodon loop of ${\text{tRNA}}_{\text{GUC}}^{\text{Asp}}$. (A) Quantification of Q in tRNA of E. histolytica trophozoites using LC-MS/MS. RNA samples were prepared from control trophozoites (T), trophozoites that were grown with queuine (q) supplemented in the culture medium (0.1 μM for 3 days) (T+q), trophozoites silenced for the expression of EhQTRT1 (siEhQTRT1), and trophozoites silenced for the expression of EhQTRT1 that were grown with queuine (siEhQTRT1+q). tRNA was purified and analyzed by LC-MS/MS to quantify Q using two transitions: *m/z* 410 → 163 (left) and *m/z* 410 → 295 (right). Data represent means ± SEMs for *N* = 12 (unpaired Student’s *t* test, *P* ≤ 0.0001). (B) Bisulfite sequencing of ${\text{tRNA}}_{\text{GUC}}^{\text{Asp}}$ was performed on control trophozoites (T), trophozoites that were grown with 0.1 μM queuine for 3 days (T+q), trophozoites silenced for the expression of EhQTRT1 (siEhQTRT1), and trophozoites silenced for the expression of EhQTRT1 that were grown with queuine (siEhQTRT1+q). The number of independent clones (sequence reads) is shown in parentheses to the left of each row. The black circles represent methylated cytosine residues, whereas the white circles represent unmethylated cytosine residues. The percentage of cytosine methylation is indicated below each circle. The location of specific cytosine residues is indicated above the circles. The level of cytosine-38 ${\text{tRNA}}_{\text{GUC}}^{\text{Asp}}$ methylation in T+q (52.5% ± 3.5%) was significantly different from that of T (20% ± 1.4%) according to the analysis with an unpaired Student’s *t* test (*P* < 0.05). Indeed, the levels of cytosine 38 ${\text{tRNA}}_{\text{GUC}}^{\text{Asp}}$ methylation in siEhQTRT1 trophozoites (siEhQTRT1) and in siEhQTRT1 trophozoites that were grown with queuine (siEhQTRT1+q) were not significantly different according to the analysis with an unpaired Student’s *t* test (*P* < 0.05). The data are expressed as means ± standard deviations (SDs) obtained from 20 clones each.

**FIG 2:**
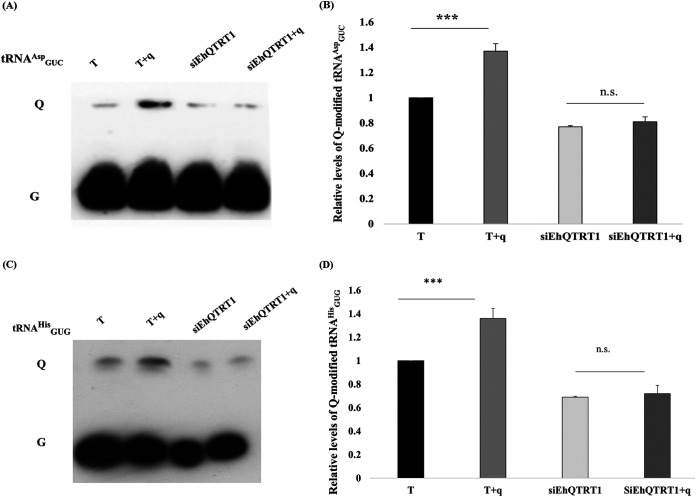
Acidic urea-PAGE analysis of ${\text{tRNA}}_{\text{GUC}}^{\text{Asp}}$ and APB Northern blot analysis of ${\text{tRNA}}_{\text{GUG}}^{\text{His}}$. (A) Acidic urea-PAGE analysis of ${\text{tRNA}}_{\text{GUC}}^{\text{Asp}}$ in E. histolytica trophozoites. (A) First lane, control trophozoites (T); second lane, control trophozoites that were grown with queuine (T+q), third lane, siEhQTRT1 trophozoites (siEhQTRT1); fourth lane, siEhQTRT1 trophozoites that were grown with queuine (siEhQTRT1+q). Total RNA samples were run on a 15% acrylamide gel supplemented with 0.1 M sodium acetate (pH 4.8), transferred to a nylon membrane, dried, and incubated with a biotinylated probe against ${\text{tRNA}}_{\text{GUC}}^{\text{Asp}}$. (B) Quantitative analysis of relative levels of Q-modified ${\text{tRNA}}_{\text{GUC}}^{\text{Asp}}$. The signal intensity of the Q-tRNA band for each sample was divided by the signal intensity for the total tRNA content (Q+G) and then normalized to the wild-type (T) samples. The data represent means ± SDs from three independent experiments (unpaired Student’s *t* test, *P* = 0.0004). (C) APB Northern blot analysis for ${\text{tRNA}}_{\text{GTG}}^{\text{His}}$. First lane, control trophozoites (T); second lane, control trophozoites that were grown with queuine (T+q); third lane, siEhQTRT1 trophozoites that were grown with queuine (siEhQTRT1+q); fourth lane, siEhQTRT1 trophozoites. Total RNA samples were run on a 15% acrylamide gel supplemented with APB, transferred to a nylon membrane, dried, and incubated with a biotinylated probe against ${\text{tRNA}}_{\text{GUG}}^{\text{His}}$. (D) Quantitative analysis of relative levels of Q-modified ${\text{tRNA}}_{\text{GUG}}^{\text{His}}$. The signal intensity of the Q-tRNA band for each sample was divided by the signal intensity for the total tRNA content (Q+G) and then normalized to the wild-type (T) samples. The data represent means ± SDs from three independent experiments (unpaired Student’s *t* test, *P* = 0.0003).

We observed that supplementation of queuine in the Trypticase-yeast extract-iron serum (TYI-S-33) growth medium caused a >5-fold increase in the level of Q in tRNAs as measured by LC-MS/MS (Fig. 1A). We also quantified the levels of Q in ${\text{tRNA}}_{\text{GUG}}^{\text{His}}$ and ${\text{tRNA}}_{\text{GUC}}^{\text{Asp}}$ by using *N*-acryloyl-3-aminophenylboronic acid (APB) polyacrylamide gel and acidic polyacrylamide gel, respectively, to separate Q-tRNAs from unmodified tRNAs (Fig. 2A and C). We observed a very strong increase of the levels of Q- ${\text{tRNA}}_{\text{GUG}}^{\text{His}}$ and Q- ${\text{tRNA}}_{\text{GUC}}^{\text{Asp}}$ in trophozoites that were grown with queuine compared to that in control trophozoites that were grown without queuine (Fig. 2B and D).

These data indicate that queuine is incorporated into E. histolytica tRNAs, suggesting the presence of an active TGT in the parasite. The results also raise concerns about the limited amount of queuine available for E. histolytica in the complete TYI-S-33 medium.

Recently, it was found that a modification of the wobble position of tRNA with a GUN anticodon by 7-deaza-guanosine derivative queuosine (Q34) stimulates S. pombe Dnmt2/Pmt1-dependent C38 methylation (m^5^C38) in the ${\text{tRNA}}_{\text{GUC}}^{\text{Asp}}$ anticodon loop (17). The presence of an active Dnmt2 in E. histolytica (26–28) incited us to check if queuine also enhances the level of m^5^C38 in the parasite’s ${\text{tRNA}}_{\text{GUC}}^{\text{Asp}}$ anticodon loop (Fig. 1B). We used PCR-based bisulfite sequencing to detect cytosine methylation in the tRNA by identifying the location of the methylated cytosine by sequencing. Amplicons were generated from trophozoites that were grown with and without queuine, and sequences of several independent PCR products were sequenced and analyzed. We observed that the percentage of C38 ${\text{tRNA}}_{\text{GUC}}^{\text{Asp}}$ methylation in trophozoites that were grown with queuine (52.5% ± 3.5%) was significantly higher than the percentage of C38 ${\text{tRNA}}_{\text{GUC}}^{\text{Asp}}$ methylation in control trophozoites (20% ± 1.4%). Moreover, we were unable to detect methylation at C-32, C-33, C-37, C-48, and C-56, whereas 100% methylation was observed at C-49. These results which are in accordance with our previously published results (26) indicate that the bisulfite reaction was successful. These results also indicate that the incorporation of queuine into amoebic tRNAs leads to hypermethylation of ${\text{tRNA}}_{\text{GUC}}^{\text{Asp}}$.

### Characterization of EhTGT.

Given the queuine-induced increase in Q in tRNA, we next set out to characterize the E. histolytica TGT (EhTGT) and its role in Q incorporation and cell phenotype. We used a homology-based approach with the human *QTRT1* gene to identify the subunits of E. histolytica TGT. The eukaryotic TGT enzymes, such as human TGT, consist of queuine tRNA-ribosyltransferase 1 (QTRT1; eubacterial TGT homolog) and queuine tRNA-ribosyltransferase domain-containing 1 (QTRTD1). To get more information about EhTGT, we performed a protein sequence alignment of Homo sapiens QTRT1 subunit (hQTRT1), E. histolytica QTRT1 (EhQTRT1), and E. histolytica QTRTD1 (EhQTRTD1) (see Fig. S2A available at https://datadryad.org/stash/share/Y6qAAIhEzUcPNlK16zSmOvPJyjd0bqUcPUz3mNw1fCg). Based on the annotation of the E. histolytica genome, homologs of hQTRT1 and hQTRTD1 exists in E. histolytica, namely, EhQTRT1 (XP_656142.1) and EhQTRTD1 (XP_652881.1). The EhQTRT1 gene shares a high degree of homology with human *QTRT1* (52% sequence identity), whereas EhQTRTD1 shares a homology at the C-terminal domain (28.3% sequence identity). Moreover, these two proteins also share a high degree of homology with the Zymomonas mobilis TGT (41.6% and 35.6%, respectively). The Z. mobilis TGT enzyme is a homodimeric zinc-binding protein, whereas the eukaryotic TGT is a heterodimeric zinc-binding protein. We identified four essential residues that are important for Zn^2+^ binding (Cys 305, Cys307, Cys310, and His342; E. histolytica numbering) to be conserved in both EhQTRT1 and EhQTRTD1 (see Fig. S2B available at https://datadryad.org/stash/share/Y6qAAIhEzUcPNlK16zSmOvPJyjd0bqUcPUz3mNw1fCg).

**FIG 3:**
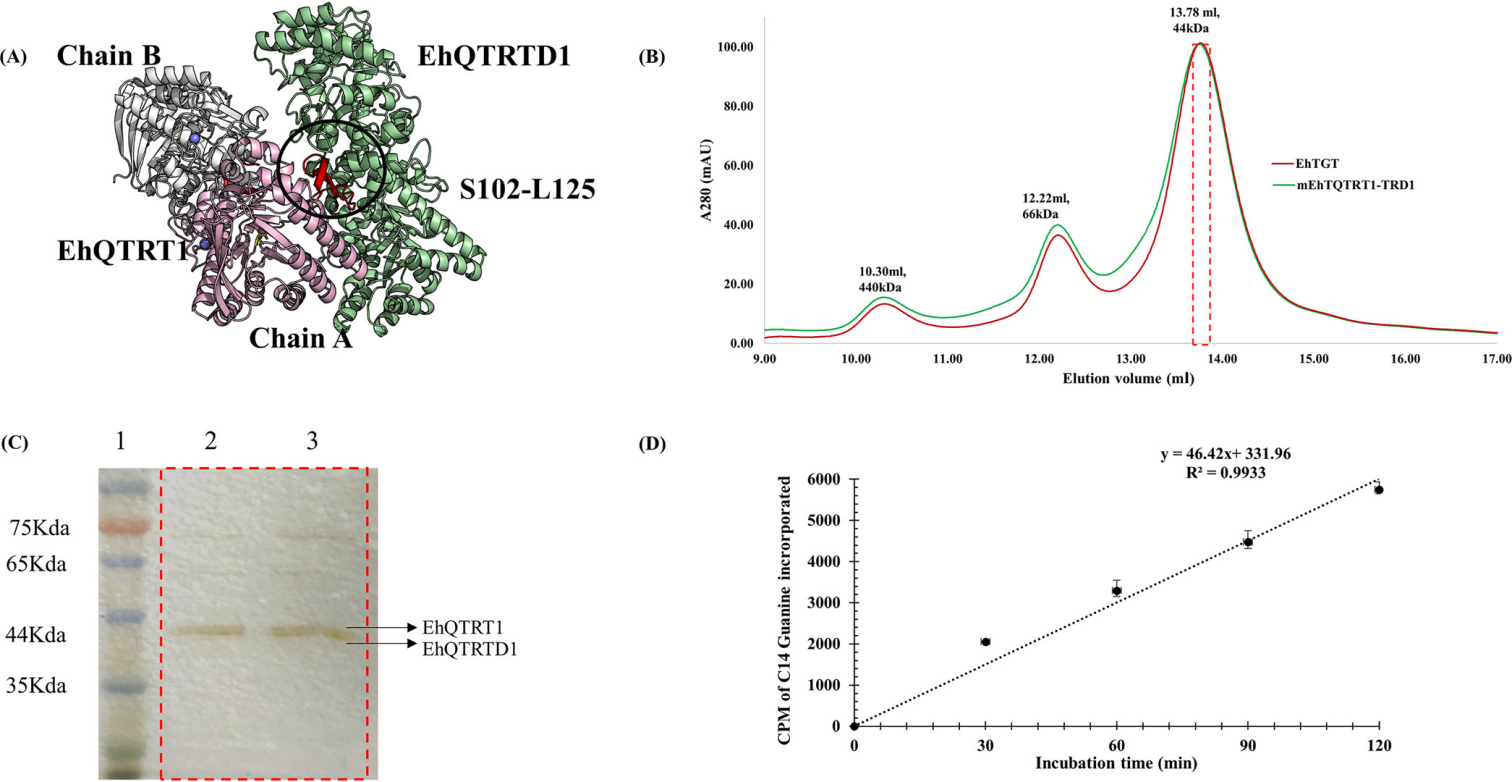
*In silico* modeling of EhTGT and expression in E. coli. (A) *In silico* model analysis showing the interaction of EhQTRT1 and EhQTRTD1 and expression of EhTGT in E. coli. The EhQTRT1-EhQTRTD1 complex was successfully modeled. EhQTRT1 and EhQTRTD1 were coexpressed in E. coli using a pETDuet-1 vector. (B) A size exclusion chromatogram representing purified EhTGT (red line) and mEhQTRT1-QTRTD1 (green line). The elution volumes of each protein are mentioned at the top of each peak along with their predicted molecular weight after elution from the column. (C) SDS-PAGE showing the presence of EhQTRT1 and EhQTRTD1 in EhTGT (lane 2) and mEhQTRT1 and EhQTRTD1 in the mutated EhTGT (lane 3). The elution volumes (shown in red box) correspond to peak obtained after size exclusion chromatography for each protein. (D) Counts per minute of 8-[^14^C]guanine incorporated over time in E. histolytica ${\text{tRNA}}_{\text{GUC}}^{\text{Asp}}$ over time in the presence of EhTGT (blue line). The graph represent means ± SDs of cpm values from three independent experiments performed in triplicates.

To get information about the EhTGT structure, we built an *in silico* model of EhTGT subunits based on the Thermotoga maritima QTRT1 structure and murine QTRT2 structures. The prediction also suggested that EhQTRT1 and EhQTRTD1 are homodimers (see Fig. S3A and B available at https://datadryad.org/stash/share/Y6qAAIhEzUcPNlK16zSmOvPJyjd0bqUcPUz3mNw1fCg), which interact together to form a heterodimer like the eukaryotic TGT enzyme (Fig. 3A) (29). To get more insights about the EhTGT enzyme, a polyhistidine-tagged EhQTRT1 and untagged EhQTRTD1 were cloned and coexpressed in E. coli. During protein expression, the addition of 100 μM ZnSO_4_ and low-temperature induction (19°C) are essential for the formation of the complex. EhQTRTD1 was copurified with the polyhistidine-tagged EhQTRT1 by Ni^2+^ affinity chromatography. EhTGT was then dialyzed in the absence of ZnSO_4_ in the dialysis buffer to induce the dissociation of EhQTRT1 from EhQTRTD1. EhTGT was further purified by size exclusion chromatography (Fig. 3B). The fraction that corresponds to the predicted molecular weight of EhQTRT1 and EhQTRTD1 was analyzed by SDS-PAGE (Fig. 3C). The molecular weights of both EhQTRT1 and EhQTRTD1 on SDS-PAGE were revealed to be around 44.2 kDa and 44.1 kDa, respectively, which matches their predicted molecular weight (30). To confirm the identity of both bands, mass spectrometry was performed on peptides obtained from tryptic digests of the 44.2-kDa and 44.1 kDa-bands excised from the SDS gel. After mapping these peptide fragments against E. coli and E. histolytica databases, our samples were confirmed to be EhQTRT1 and EhQTRTD1 (see Fig. S3C available at https://datadryad.org/stash/share/Y6qAAIhEzUcPNlK16zSmOvPJyjd0bqUcPUz3mNw1fCg).

To verify that the EhTGT is active, the activity of the complex was tested by determining its ability to displace guanine in the ${\text{tRNA}}_{\text{GUC}}^{\text{Asp}}$ anticodon with 8-[^14^C]guanine. We observed that EhTGT is an active enzyme and that the EhQTRT1-EhQTRTD1 enzyme complex efficiently incorporated 8-[^14^C]guanine with a specific activity of 2.28 pmol/min/mg of protein (Table 1) (Fig. 3D). However, no activity was detected when ${\text{tRNA}}_{\text{CAC}}^{\text{Val}}$ was used as a substrate (Table 1).

**TABLE 1:** Validation of the *in silico* model of EhTGT^*a*^

Enzyme	Substrate	Sp act of the enzyme (pmol/min/mg of protein)
EhTGT	${\text{tRNA}}_{\text{GUC}}^{\text{Asp}}$	2.28 ± 0.01
mEhQTRT1-TRTD1	${\text{tRNA}}_{\text{GUC}}^{\text{Asp}}$	0.01 ± 0.02
EhTGT	${\text{tRNA}}_{\text{CAC}}^{\text{Val}}$	0

a Our results indicate that EhTGT is an active enzyme when it forms a functional complex and when ${\text{tRNA}}_{\text{GUC}}^{\text{Asp}}$ is used as the substrate. A point mutation (D267 to A267) in the predicted active site of EhQTRT1 makes the enzyme (mEhQTRT1-TRTD1) inactive without affecting the formation of the EhQTRT1-EhQTRTD1 complex. No EhTGT activity was detected when ${\text{tRNA}}_{\text{CAC}}^{\text{Val}}$ was used as the substrate. The specific activity of EhTGT incubated with ${\text{tRNA}}_{\text{GUC}}^{\text{Asp}}$ was significantly different from mEhQTRT1-EhQTRTD1 activity and from EhTGT activity when the enzyme was incubated with ${\text{tRNA}}_{\text{CAC}}^{\text{Val}}$ according to an unpaired Student’s *t* test (*P* ≤ 0.05). The data are expressed as means ± standard deviations and are representative of three independent experiments performed in duplicates.

The *in silico* model also predicted that the stretch of S102 to L125 in EhQTRT1 is essential for the binding of EhQTRTD1, and that the D267 residue in EhQTRT1 is a substrate-binding residue (Fig. 3A). A point mutation (D267 to A267) in the predicted active site of EhQTRT1 (indicated in a black circle in Fig. 3A) made the enzyme inactive without affecting the formation of the EhQTRT1-EhQTRTD1 complex (Table 1; Fig. 3C). These results confirm the prediction of the *in silico* model regarding the location of the active site (Fig. 3A).

**FIG 4:**
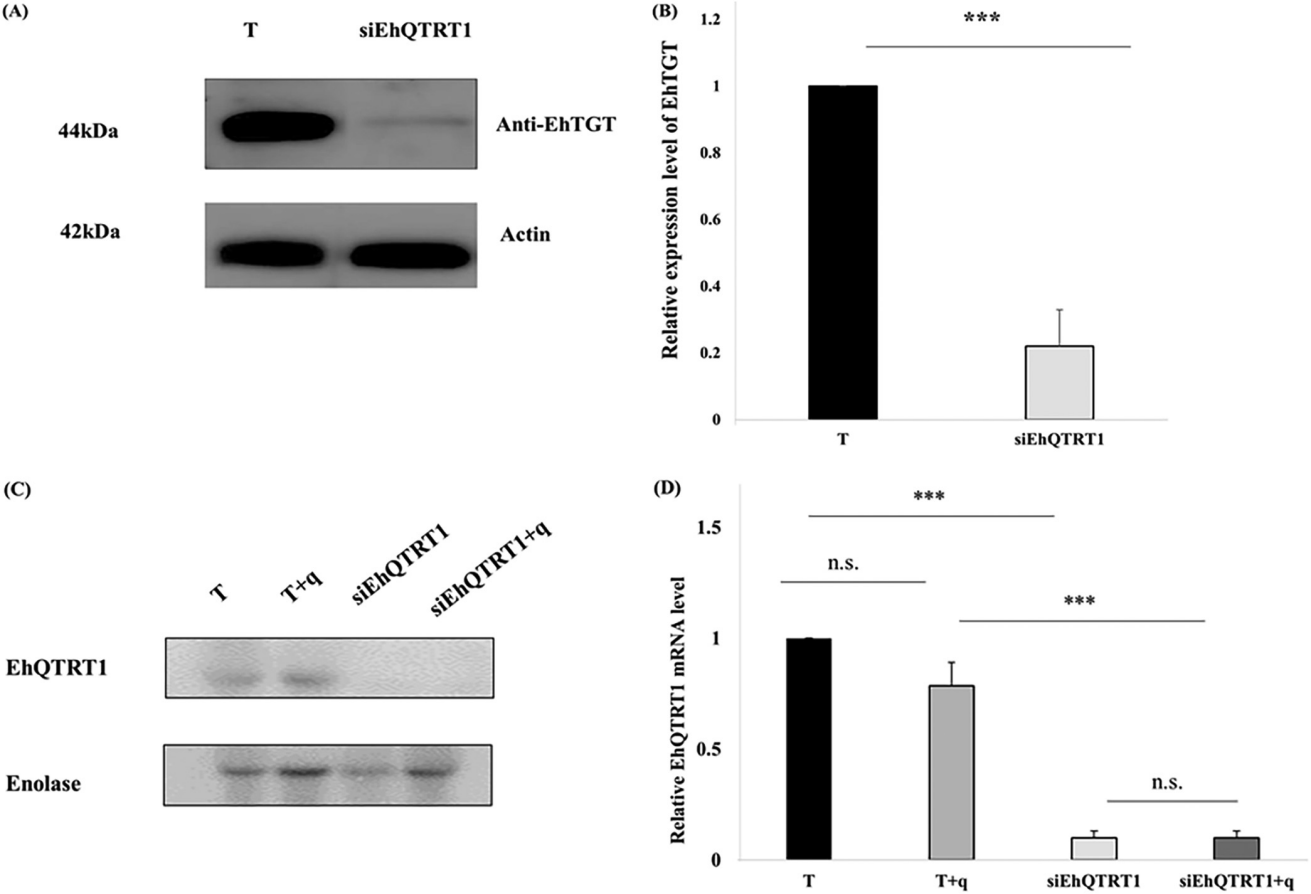
Silencing of EhTGT. (A) Western blotting was performed on total protein extracts that were prepared from wild-type E. histolytica trophozoites (T) and EhQTRT1-silenced trophozoites. The proteins were separated on 10% SDS-PAGE gels and analyzed by Western blotting using a homemade EhTGT antibody (1:1,000) or actin antibody (1:1,000). (B) Densitometry analysis showing the percentage of inhibition of the EhTGT complex in EhQTRT1-silenced trophozoites (normalized to wild type). The data represent means ± SDs from three independent experiments (unpaired Student’s *t* test, *P* = 0.0003). (C) Northern blot analysis of EhQTRT1. Enolase was used as the housekeeping gene because its expression does not change in response to queuine (this work). (D) Quantitative analysis of relative density of mRNA. The signal intensities of the samples were divided by enolase mRNA and were then normalized to the wild-type mRNA. Data are presented as means ± standard deviations from two independent experiments.

**FIG 5:**
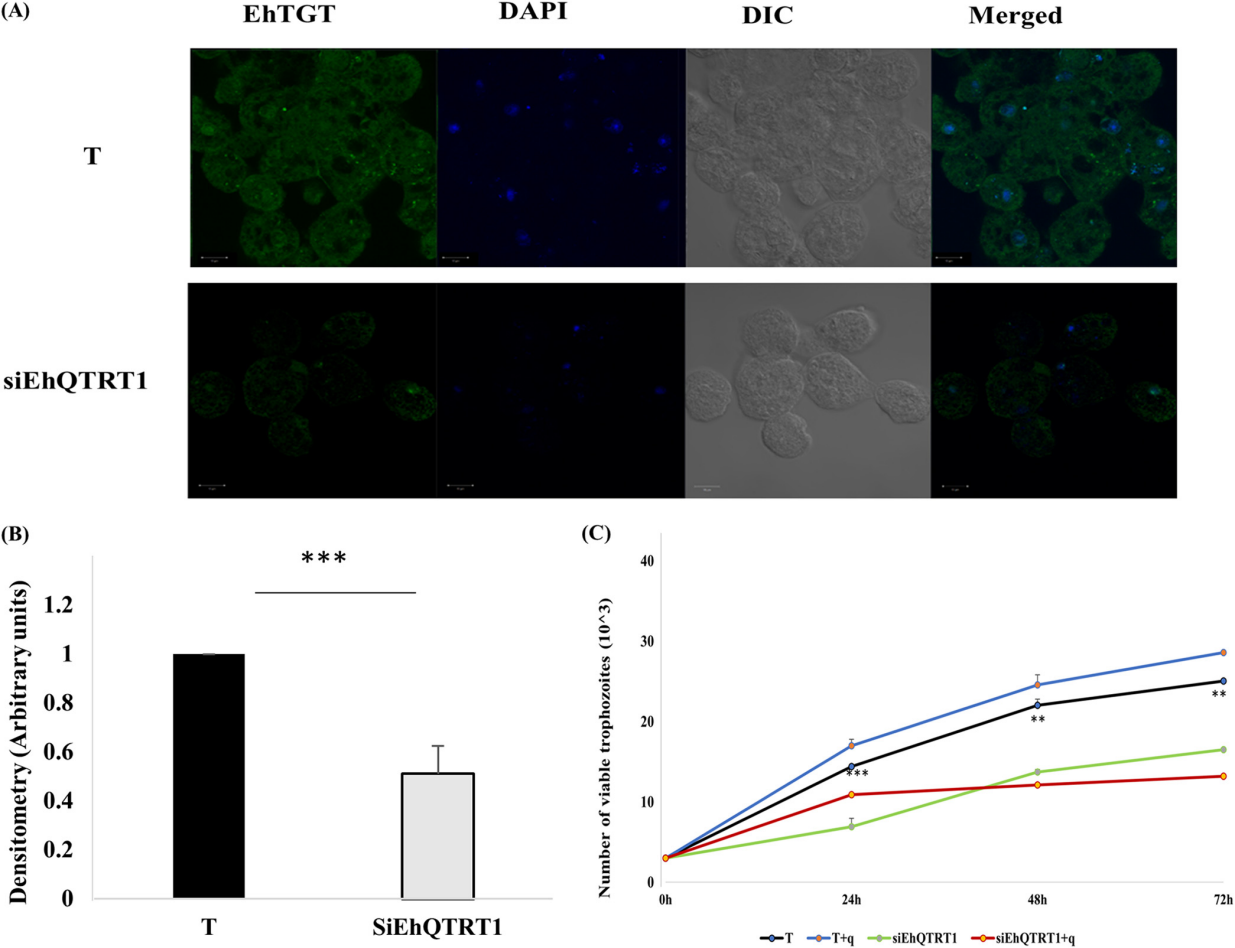
Characterization of the EhTGT-silenced trophozoites. (A) EhTGT was detected in control trophozoites (T) and siEhQTRT1 trophozoites (siEhQTRT1) using a homemade polyclonal mouse EhTGT antibody (1:250) and then incubated with a goat anti-mouse secondary antibody (Alexa Fluor; Jackson Immuno Research) at a concentration of 1:250. Nuclei are stained with 4′,6-diamidino-2-phenylindole (DAPI) (Sigma-Aldrich) at concentration of 1:1,000. The samples were examined under a confocal immunofluorescence microscope (Zeiss LSM700 Meta laser scanning confocal imaging system) with a 63× oil immersion lens objective. Images are scaled to 10 μm. (B). Densitometry analysis showing the percentage of the EhTGT complex in 15 trophozoites under each condition (normalized to control trophozoites). Data are expressed as means ± SDs from three independent experiments (unpaired Student’s *t* test, *P* ≤ 0.0001). (C) Effect of silencing EhQTRT1 on the growth of E. histolytica. Control trophozoites (T) and trophozoites silenced for EhQTRT1 (siEhQTRT1) (30 × 10^3^/ml of complete TYI medium) were grown for 24 h, 42, and 72 h with queuine (+q) or without queuine. The number of viable trophozoites at each time point was determined by using the vital stain eosin. siEhQTRT1 trophozoites have their growth impaired compared to that of control trophozoites. Queuine has no significant effect on the growth of T or SiEhQTRT1 trophozoites. The data are representative of three independent experiments performed in duplicates. Data are expressed as means ± SDs. (unpaired Student’s *t* test, T versus siEhQTRT1 24 h [*P* = 0.0006], 48 h [*P* = 0.0082], 72 h [*P* = 0.005]).

To better understand the role of EhTGT in E. histolytica, we silenced the expression of EhQTRT1 by using an epigenetic method based on antisense small RNAs (31). In this method, a gene coding region to which large numbers of antisense small RNAs map is used as a “trigger” to silence the gene fused to it. The silencing is maintained after the trigger plasmid has been cured of the silenced strain. Here, we used the small interfering EhQTRT1 (siEhQTRT1) plasmid to silence the expression of EhQTRT1. Following transfection of trophozoites with the siEhQTRT1 plasmid and the selection of a population resistant to G418 (6 μg/ml), the siEhQTRT1 plasmid was cured by removal of G418 from the culture for 1 month. Silencing of EhQTRT1 in the cured EhQTRT1-silenced trophozoites was confirmed by immunoblotting using an antibody against EhTGT (Fig. 4A) and by Northern blotting by using a radiolabeled probe against EhQTRT1 (Fig. 4C). Using densitometry analysis, we observed that the EhTGT complex in siEhQTRT1 trophozoites was inhibited by 80% at the protein level (Fig. 4B) and by 90% at the mRNA level (Fig. 4D). The cellular localization of EhTGT in control trophozoites and siEhQTRT1 trophozoites was performed by confocal immunofluorescence microscopy. In control trophozoites, EhTGT had a cytoplasmic localization which was barely detectable in siEhQTRT1 trophozoites (Fig. 5A and B).

We have previously demonstrated that EhTGT can incorporate queuine into E. histolytica tRNAs (Fig. 1A). Consequently, we assumed that siEhQTRT1 trophozoites would have a reduced ability to incorporate queuine in their tRNAs. To test this assumption, we determined the global levels of Q in siEhQTRT1 trophozoites tRNAs (Fig. 1A) and in Q- ${\text{tRNA}}_{\text{GUG}}^{\text{His}}$ and ${\text{tRNA}}_{\text{GUC}}^{\text{Asp}}$ (Fig. 2A and B). We observed a very strong reduction of the levels of Q-tRNAs, Q- ${\text{tRNA}}_{\text{GUG}}^{\text{His}}$, and ${\text{tRNA}}_{\text{GUC}}^{\text{Asp}}$ in siEhQTRT1 trophozoites that were grown with queuine compared to that in control trophozoites that were grown with queuine. The amount of m^5^C38 methylation in ${\text{tRNA}}_{\text{GUC}}^{\text{Asp}}$ in siEhQTRT1 trophozoites that were grown with queuine was also significantly reduced (23.5% ± 2.1%) compared to the level in control trophozoites that were grown with queuine (52.5% ± 3.5%) (Fig. 1B).

We also observed that siEhQTRT1 trophozoites had their growth impaired by 50% compared to the growth rate of the control trophozoites (Fig. 5C). Queuine had no significant effect on the growth of control or siEhQTRT1 trophozoites (Fig. 5C).

### Queuine induces the resistance of E. histolytica to OS.

**FIG 6:**
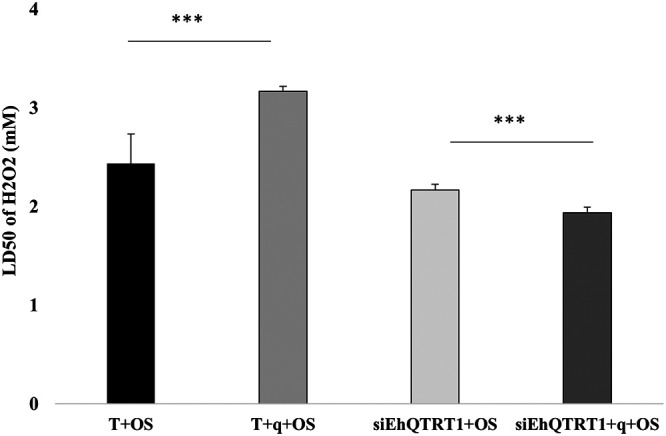
Queuine triggers resistance to OS, and this effect is lost in siEhQTRT1 trophozoites. Determination of the amount of H_2_O_2_ required to kill 50% of the population (LD_50_) of control trophozoites, trophozoites that were grown with queuine (0.1 μM for 3 days), siEhQTRT1 trophozoites, and siEhQTRT1 trophozoites cultivated with queuine. The data represent means ± SDs from three independent experiments performed in duplicates. The LD_50_ of trophozoites that were grown with queuine and exposed to OS (T+q+OS) was significantly higher than the LD_50_ of untreated trophozoites exposed to OS (T) (unpaired Student’s *t* test, *P* ≤ 0.005). The LD_50_ of siEhQTRT1 trophozoites that were grown with queuine and exposed to OS (siEhQTRT1+q+OS) was significantly lower than the LD_50_ of siEhQTRT1 trophozoites exposed to OS (siEhQTRT1+OS) (unpaired Student’s *t* test, *P* ≤ 0.005).

Having established that queuine supplementation leads to increased Q levels in tRNA, we next defined the effect of queuine on the parasite phenotype. To understand if queuine regulates resistance to OS in the parasite, we grew trophozoites with and without queuine and exposed them to different concentrations of H_2_O_2_ (0 to 5 mM). We observed that the 50% lethal dose (LD_50_) of H_2_O_2_ for trophozoites that were grown with queuine (3.3 ± 0.05 mM) was significantly higher than in the absence of queuine (2.4 ± 0.3 mM) (Fig. 6). These results indicate that queuine protects the parasite against OS. Next, we asked whether the beneficial effect that queuine has on the resistance of E. histolytica to OS (Fig. 6) depends on EhTGT. For this purpose, we determined the resistance of siEhQTRT1 trophozoites that were grown with queuine and then exposed to OS (Fig. 6). We observed that queuine did not protect siEhQTRT1 trophozoites against OS.

### Queuine regulates protein synthesis during OS.

**FIG 7:**
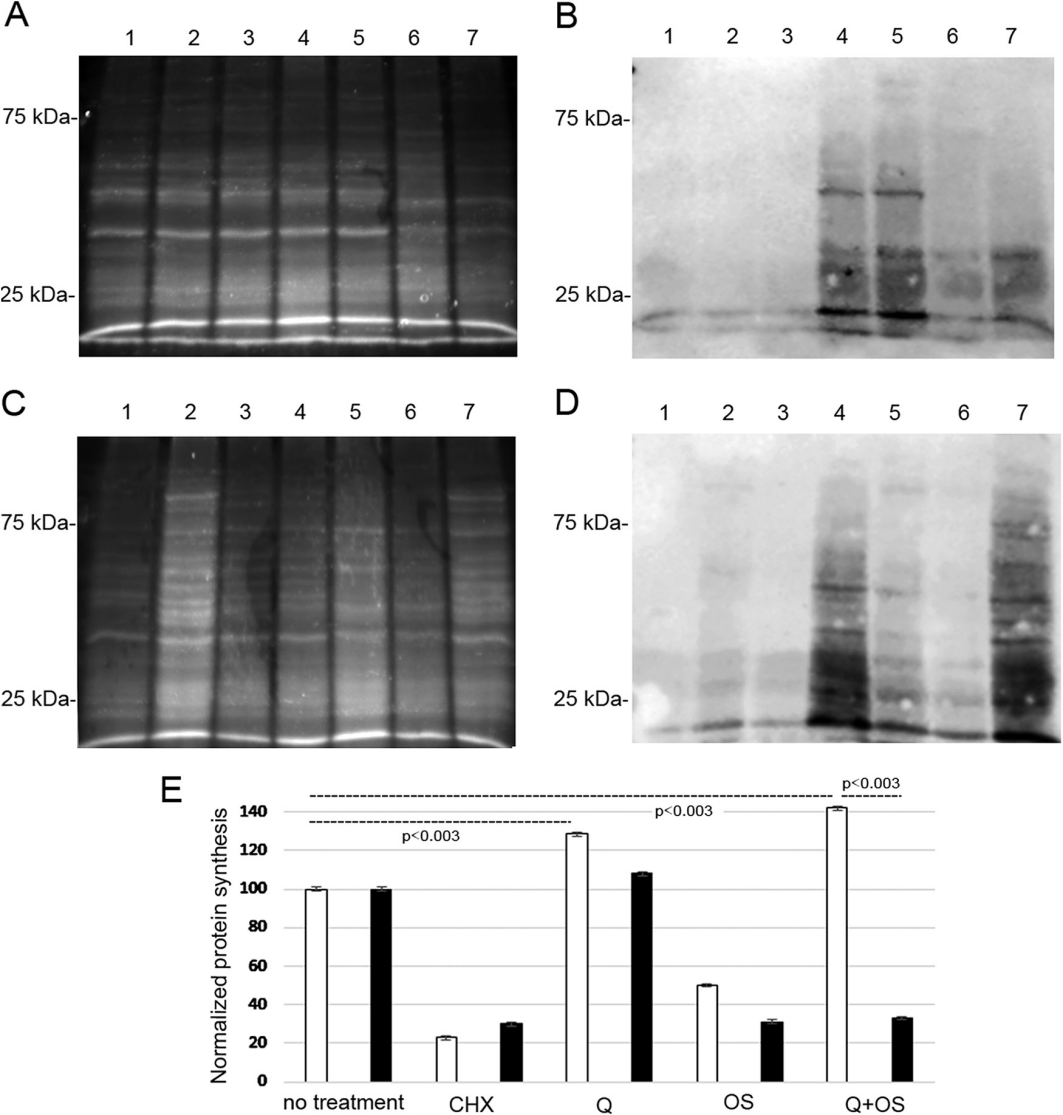
Measurement of protein synthesis in control trophozoites and in siEhQTRT1 trophozoites using puromycin-labeled proteins. (A and C) Stain-free total protein labeling. (B and D) Western blot with puromycin antibody. (A and B) Lane 1, control trophozoites; lane 2, control trophozoites that were grown with queuine (0.1 μM for 3 days); lane 3, control trophozoites treated with cycloheximide (100 μg/ml) before being labeled with puromycin (10 μg/ml); lane 4, control trophozoites labeled with puromycin; lane 5, control trophozoites that were grown with queuine before being labeled with puromycin; lane 6, control trophozoites exposed to 2.5 mM H_2_O_2_ for 20 min before being labeled with puromycin; lane 7, trophozoites that were grown with queuine and then exposed to 2.5 mM H_2_O_2_ before being labeled with puromycin. (C and D) Lane 1, siEhQTRT1 trophozoites; lane 2, siEhQTRT1 trophozoites that were grown with queuine (0.1 μM for 3 days); lane 3, siEhQTRT1 trophozoites treated with cycloheximide (100 μg/ml) before being labeled with puromycin (10 μg/ml); lane 4, siEhQTRT1 trophozoites labeled with puromycin; lane 5, siEhQTRT1 trophozoites that were grown with queuine prior to their exposure to 2.5 mM H_2_O_2_ before being labeled with puromycin; lane 6, siEhQTRT1 trophozoites exposed to 2.5 mM H_2_O_2_ before being labeled with puromycin; lane 7, siEhQTRT1 trophozoites that were grown with queuine before being labeled with puromycin. (E) Normalization with ImageJ of the puromycin signal (B) against the total protein signal (A) for control trophozoites (white bars) and of the puromycin signal (D) against the total protein signal (C) for siEhQTRT1 trophozoites (black bars). Normalized values for control trophozoites labeled with puromycin and for siEhQTRT1 trophozoites labeled with puromycin were taken as 100%. Total protein extracts were separated on an SDS-PAGE gel, and they were analyzed by Western blotting with a mouse-monoclonal puromycin antibody (1:5,000). The data represent means ± SDs from two independent experiments (unpaired Student’s *t* test, *P* < 0.003). Total protein was determined using stain-free detection.

The protective effect that queuine has on parasites exposed to OS suggests that protein synthesis is preserved in the parasites exposed to queuine and OS. To test this hypothesis, we used the surface sensing of translation assay (SUnSET) (32) to determine the amount of puromycin that was incorporated into nascent peptide chains (Fig. 7A, B, and E). As previously described (33), we found that protein synthesis was strongly inhibited in trophozoites exposed to cycloheximide, a protein synthesis inhibitor, and in trophozoites exposed to OS (Fig. 7B and E). The inhibitory effect of OS on protein synthesis did not occur in trophozoites that were grown with queuine before being exposed to OS (Fig. 7B and E). Interestingly, queuine seems to improve the level of protein synthesis in control trophozoites (Fig. 7B and E). Next, we asked whether the regulatory effect that queuine has on protein synthesis depends on EhTGT. For this purpose, the SUnSET assay was performed on siEhQTRT1 trophozoites that were grown with and without queuine and then exposed or not to OS (Fig. 7C, D, and E). None of the effect that queuine has on protein synthesis was observed in siEhQTRT1 trophozoites.

### Queuine impairs cytopathic activity and the survival of the parasite within the mouse cecum.

**FIG 8:**
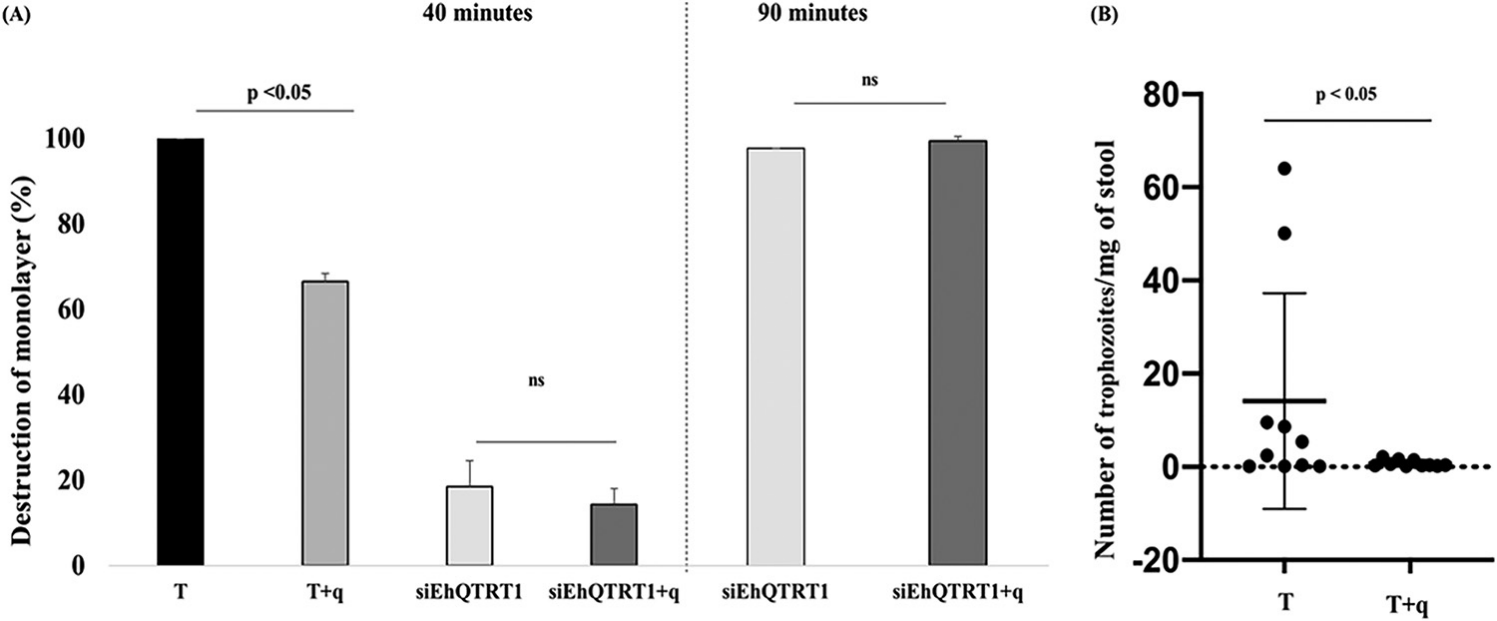
Effect of queuine on the virulence of E. histolytica. (A). Cytopathic activity of E. histolytica trophozoites and siEhQTRT1 trophozoites that were grown with and without queuine was measured by their ability to destroy a monolayer of HeLa cells. E. histolytica trophozoites were grown with and without queuine (0.1 μM for 3 days). Data are displayed as the means ± standard deviations from three independent experiments that were repeated twice (unpaired Student’s *t* test; T versus T+q, *P* = 0.0026). siEhQTRT1 trophozoites that were grown with and without queuine were incubated with a HeLa cell monolayer for 40 or 90 min. The cytopathic activity of siEhQTRT1 trophozoites that were grown with queuine is not different from the cytopathic activity of siEhQTRT1 trophozoites that were grown without queuine at both 40 and 90 min of incubation between the trophozoites and the HeLa cell monolayer. The data are representative of three independent experiments (normalized to wild type) (unpaired Student’s *t* test, *P* = 0.01 [T versus T+q]). (B) Effects of queuine treatment in intestinal amoebiasis. Number of trophozoites determined in stool samples from CBA/J mice infected with trophozoites that were grown with queuine (q) or with nontreated trophozoites (T). Trophozoite number was quantified by the quantitative real-time PCR method using E. histolytica 18S rRNA-specific primers. Data represent the means ± SDs from 10 mice. *P* < 0.05 by an unpaired Student’s *t* test.

It was previously shown that TGT and presumably queuine regulate the virulence of *Shigella* by controlling the translation of VirF, a central transcriptional regulator of virulence factors involved in cellular invasion and the spreading of this bacterium (34). To test whether queuine affects the virulence of the parasite, we determined the ability of E. histolytica trophozoites that were grown with and without queuine to destroy a monolayer of mammalian cells (cytopathic activity) (Fig. 8A). We found that the cytopathic activity of trophozoites that were grown in the presence of queuine was impaired compared to that of trophozoites that were grown in the absence of queuine.

Next, we asked whether the detrimental effect that queuine has on the cytopathic activity of E. histolytica (Fig. 8A) depends on EhTGT. We found that the cytopathic activity of siEhQTRT1 trophozoites was significantly impaired (Fig. 8A) compared to that of control trophozoites (Fig. 8A). Moreover, the growth of siEhQTRT1 trophozoites in the presence of queuine did not have any significant effect on their cytopathic activity (Fig. 8A). To determine if the siEhQTRT1 trophozoites require more time to destroy the HeLa monolayer than control trophozoites, we incubated siEhQTRT1 trophozoites that were grown with and without queuine on the HeLa cell monolayer for 90 min instead of 40 min. We found that more than 90% of the HeLa monolayer was destroyed by siEhQTRT1 trophozoites after 90 min of incubation (Fig. 8A). The same level of monolayer destruction was observed for siEhQTRT1 trophozoites that were grown with queuine (Fig. 8A). These results indicate that queuine impairs the strong cytopathic activity of control trophozoites (Fig. 8A) but does not impair the weak cytopathic activity of siEhQTRT1 trophozoites.

To validate the alteration of amoebic pathogenicity *in vivo*, trophozoites that were grown in the presence of queuine were subjected to mouse intestinal amoebiasis (Fig. 8B). In the mouse intestinal amebiasis model, control trophozoites or trophozoites that were grown in the presence of queuine were inoculated into susceptible CBA/J mouse cecum. At 7 days postinoculation, the number of trophozoites present in the stool was determined. The average number of detectable trophozoites from mice inoculated with control trophozoites (14 ± 23 trophozoites/mg stool) was higher than that of trophozoites that were grown in the presence of queuine (0.74 ± 0.71 trophozoites/mg stool) (Fig. 8B). These results indicate that queuine affects the survival of the parasite in the mouse cecum.

### Queuine has a profound impact on the E. histolytica transcriptome.

**FIG 9:**
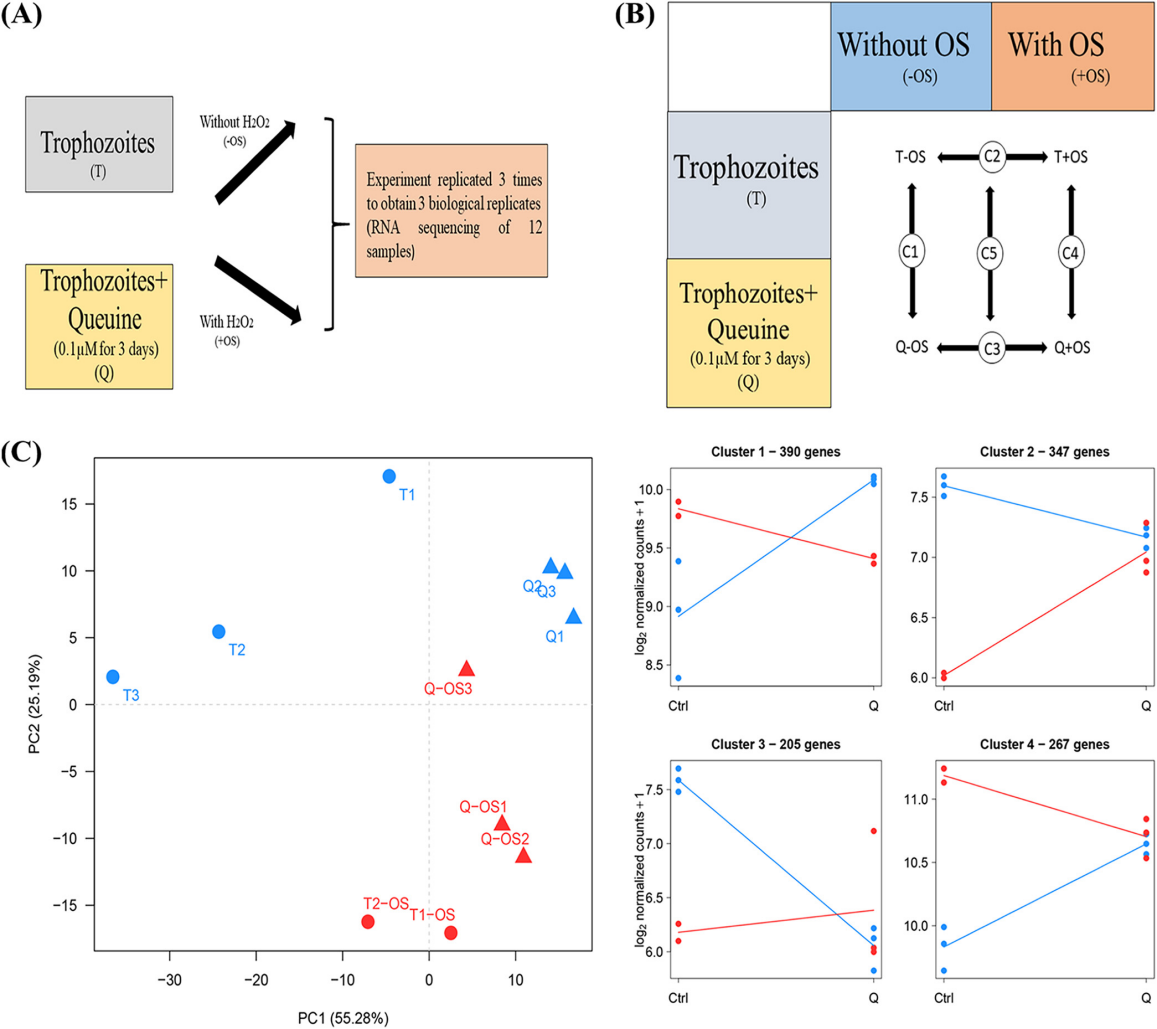
Experimental design for RNA-Seq. (A) Trophozoites were grown with and without 0.1 μM queuine for 3 days (T and q) and then were exposed or not to OS (+OS or –OS, respectively). The experiments were repeated three times to obtain 12 samples that were purified and sequenced further. (B) Biological conditions compared during differential RNA-Seq analysis. Five pairwise comparisons have been performed. (C) Principal-component analysis of the RNA-Seq data performed on the variance-stabilizing transformed count matrix. Average profiles for the four gene clusters defined by the hierarchical clustering. The average transcriptome profile was established by computing the mean log_2_-normalized count for the genes in each of the four identified clusters.

We used RNA sequencing (RNA-seq) to investigate the molecular basis of the effect of queuine on the resistance of E. histolytica to OS (Fig. 9A and B). RNA-Seq experiments were performed as described previously (10), and the data (available at http://www.ncbi.nlm.nih.gov/geo under accession number GSE142211) were analyzed using the generalized linear model implemented in the DESeq2 package in R. This allowed us to perform pairwise comparisons of gene expression by the E. histolytica HM1:IMSS strain under the various tested conditions and to probe the interaction between queuine and OS (Fig. 9B).

We first used a principal-component analysis to explore the data structure (Fig. 9C); this showed that the untreated trophozoite group (T) and the trophozoites that were grown with queuine (Q) form two distinct clusters that can be separated on the first principal component. The stress effect was mainly supported by the second component, and OS-treated and -untreated samples formed clusters under both T and Q conditions. Our comparisons revealed that queuine has a profound impact on the E. histolytica transcriptome (C1), with 664 downregulated and 548 upregulated genes (Table 2). Details regarding these genes can be found in Table S1 available at https://datadryad.org/stash/share/Y6qAAIhEzUcPNlK16zSmOvPJyjd0bqUcPUz3mNw1fCg.

**TABLE 2:** Number of genes modulated in E. histolytica control trophozoites and trophozoites that were grown with queuine in response to OS

Control	Comparison^*a*^	No. of modulated genes		
		Downregulated	Upregulated	Total
C1	Q-OS vs T-OS	664	548	1,212
C2	T+OS vs T-OS	985	661	1,646
C3	Q+OS vs Q-OS	248	92	340
C4	Q+OS vs T+OS	176	265	441
C5	C3 vs C2	485	447	932

a T, trophozoite; Q, queuine.

### Gene categories affected by queuine supplementation.

**FIG 10:**
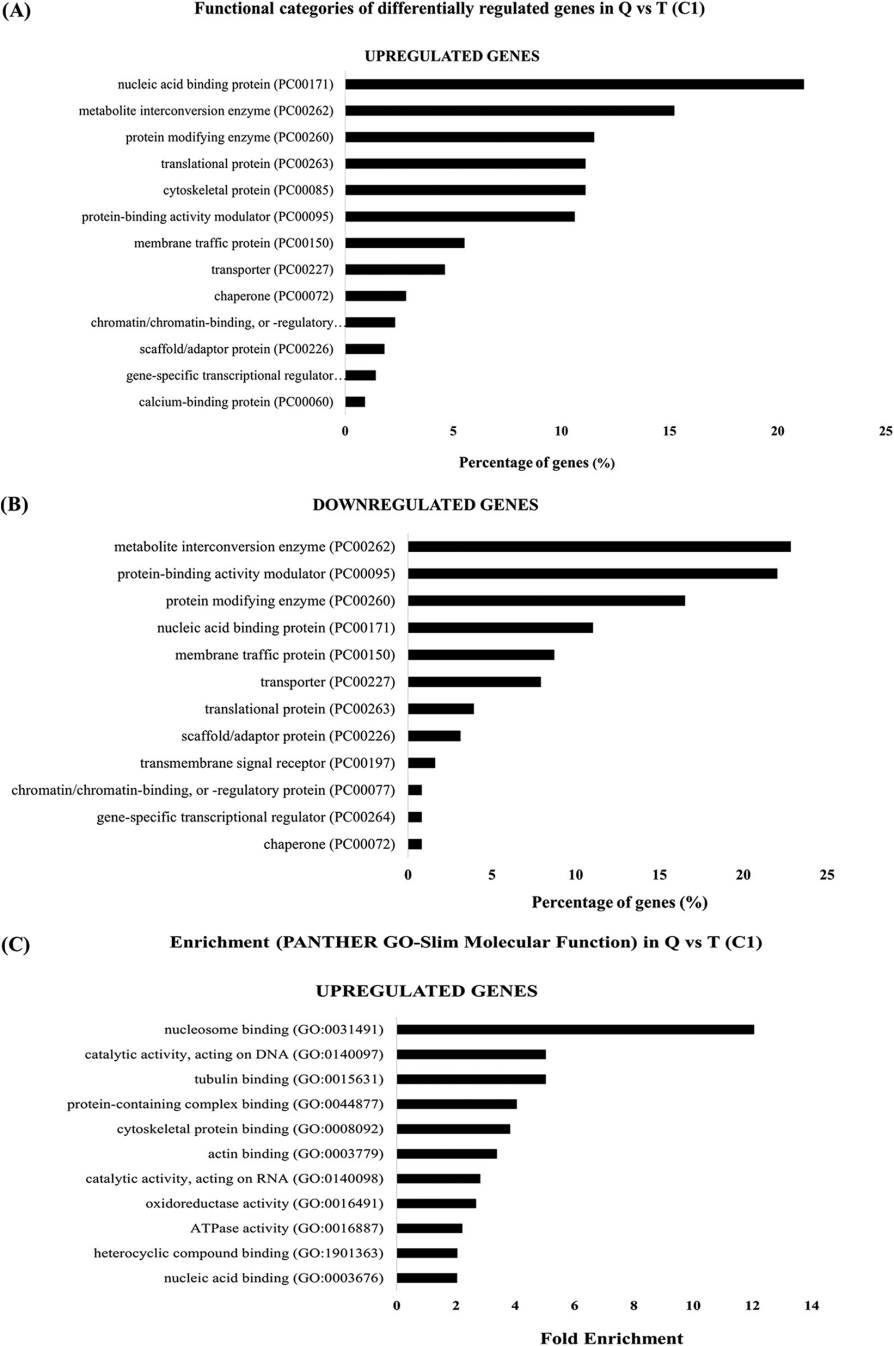
Functional categories and classification of genes enriched in trophozoites that were grown with queuine versus control trophozoites. Functional categories are upregulated genes in trophozoites that were grown with queuine (A) and downregulated genes in trophozoites that were grown with queuine (B). The number of genes related to each category was derived by processing the data (see Table S1 available at https://datadryad.org/stash/share/Y6qAAIhEzUcPNlK16zSmOvPJyjd0bqUcPUz3mNw1fCg) with PANTHER (http://pantherdb.org). (C) Enrichment test in queuine-treated trophozoites. The details of genes present in each cluster are given in Table S3 available at https://datadryad.org/stash/share/Y6qAAIhEzUcPNlK16zSmOvPJyjd0bqUcPUz3mNw1fCg.

The differentially regulated genes in Q versus T (comparison C1) were classified according to the protein class which they encode (Fig. 10A and B) using the Protein ANalysis THrough Evolutionary Relationship (PANTHER) sequence classification tool (version 14.1) (35). Of the upregulated genes in the presence of queuine (Fig. 10A), genes that are associated with the following were significantly enriched according to the PANTHER statistical overrepresentation test: nucleic acid binding (GO:0003676) and nucleosome binding (GO:0031491) such as chromatin-specific transcription elongation factor (EHI_109860) and protein HIRA (EHI_131950); heterocyclic compound binding (GO:1901363) such as 60S ribosomal protein L4 (EHI_064710) and ATP-dependent DNA helicase (EHI_119290); ATPase activity (GO:0016887) such as heat shock protein 70 family (EHI_001950) and RecQ family helicase (EHI_028890); oxidoreductase activity (GO:0016491) such as peroxiredoxin (EHI_145840) and aldehyde-alcohol dehydrogenase 2 (EHI_024240); catalytic activity, acting on RNA (GO:0140098) such as regulator of nonsense transcripts (EHI_035550) and RNA lariat debranching enzyme (EHI_062730); actin binding (GO:0003779) such as coronin (EHI_122800) and villidin (EHI_150430); cytoskeletal protein binding (GO:0008092) and tubulin binding (GO:0015631) such as dynamin-1-like protein (EHI_013180) and kinesin-like protein (EHI_140230); protein-containing complex binding (GO:0044877) such as elongation factor 2 (EHI_189490) and chromatin-specific transcription elongation factor (EHI_109860); and catalytic activity, acting on DNA (GO:0140097) such as DNA replication licensing factor (EHI_076880) and RecQ family helicase EHI_028890 (Fig. 10C).

The categories for functional classification of genes downregulated in trophozoites that were grown with queuine (using the subset of protein class) are shown in Fig. 10B. The most abundant classes of proteins were metabolite interconversion enzyme (PC00262) (22.8%), protein-binding activity modulators (PC00095) (22%), protein modifying enzymes (PC00260) (16.5%), nucleic acid binding protein (PC00171 and PC00262) (11%), membrane traffic protein (PC00150) (8.7%), transporter (PC00227) (7.9%), translational protein (PC00263) (3.9%), and scaffold/adaptor protein (PC00226) (3.1%).

Of the downregulated genes in Q, no enrichment of a specific biological process was detected according to the PANTHER statistical overrepresentation test (Fig. 10B).

### Gene categories affected by queuine supplementation and OS.

As previously described (10), OS strongly modulates the transcriptome of the parasite with 1,646 genes that are differentially expressed compared to those under the T condition (comparison C2). Interestingly, in the presence of queuine, OS affected the expression of only 340 genes, suggesting that queuine influences the response of the parasite to OS. Next, we addressed the influence of queuine on E. histolytica’s response to OS (comparison C5). We identified 932 genes (Table 2) and then performed a hierarchical clustering analysis (Fig. 9C). The average profile of each of the four identified clusters was then established (Fig. 9C) (see Table S3 available at https://datadryad.org/stash/share/Y6qAAIhEzUcPNlK16zSmOvPJyjd0bqUcPUz3mNw1fCg). Clusters 1 and 4 represent genes that were mostly upregulated in trophozoites exposed to OS but that were either mostly not differentially expressed (cluster 4) or downregulated (cluster 1) in trophozoites that were grown with queuine and exposed to OS. Clusters 2 and 3 represent genes that were mostly downregulated in control trophozoites exposed to OS but that were mostly not differentially expressed in trophozoites that were grown with queuine exposed to OS. A detailed analysis of these clusters is described below.

### Hierarchical clustering analysis.

Cluster 1 comprised 390 genes; 60.5% of these genes had their expression upregulated in control trophozoites exposed to OS versus expression in trophozoites not exposed to OS. In trophozoites that were grown with queuine and exposed to OS versus trophozoites that were grown with queuine but not exposed to OS, 48% of the genes present in cluster 1 had their expression downregulated and 52% were not differentially expressed (Table 3).

**TABLE 3:**
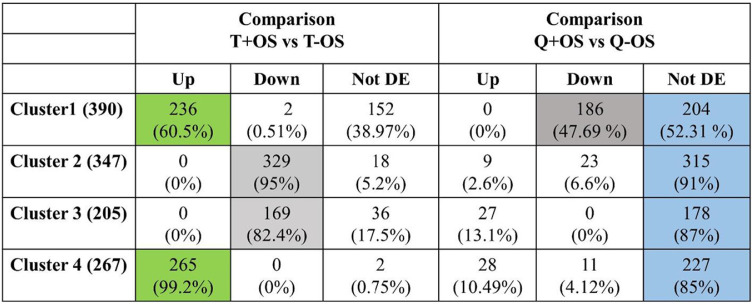
Summary of the hierarchical clustering analysis

Cluster 2 comprised 347 genes; 95% of these genes had their expression downregulated in control trophozoites exposed to OS versus that in trophozoites not exposed to OS. In trophozoites that were grown with queuine and exposed to OS versus trophozoites that were grown with queuine but not exposed to OS, 91% of the genes present in cluster 2 were not differentially expressed (Table 3).

Cluster 3 comprised 205 genes; 82.5% of these genes had their expression downregulated in control trophozoites exposed to OS versus that in trophozoites not exposed to OS. In trophozoites that were grown with queuine and exposed to OS versus trophozoites that were grown with queuine but not exposed to OS, 87% of the genes present in cluster 3 were not differentially expressed (Table 3).

Lastly, 99% of the 267 genes in cluster 4 were upregulated in control trophozoites exposed to OS versus expression in trophozoites not exposed to OS, and 85% were not differentially expressed in trophozoites that were grown with queuine and exposed to OS versus that in trophozoites that were grown with queuine but not exposed to OS (Table 3).

### Functional categories identified in queuine and OS treatments.

The analytical tools in PANTHER (http://pantherdb.org/) were used for functional classification and gene set overrepresentation tests. To identify the functional categories, we used the PANTHER tools, which include entries from E. histolytica for functional classification. The categories for functional classification within each cluster (using the subset of protein class) are shown in Fig. S4 and Table S4 available at https://datadryad.org/stash/share/Y6qAAIhEzUcPNlK16zSmOvPJyjd0bqUcPUz3mNw1fCg. The most abundant classes of proteins in cluster 1 were nucleic acid binding (PC00171) (20.3%), enzyme modulator (PC00095) (16.4%), and hydrolase (PC00121) (16.4%) (see Fig. S4A).

The most abundant classes of proteins in cluster 2 were enzyme modulator (PC00095) (22%), hydrolase (PC00121) (17%), and nucleic acid binding (PC00171) (14%) (Fig. S4B).

The most abundant classes of protein in cluster 3 were nucleic acid binding (PC00171) (21%), hydrolase (PC00121) (19%), and transferase (PC00220) (15%) (Fig. S4C).

Finally, the most abundant classes of protein in cluster 4 were nucleic acid binding (PC00171) (20.3%), hydrolase (PC00121) (19%), and enzyme modulator (PC00095) (13%) (Fig. S4D).

### Enrichment of molecular functions.

We restricted our analyses to the families of factors enriched >2.0-fold in the overrepresentation test (see Fig. S5 and Table S4 available at https://datadryad.org/stash/share/Y6qAAIhEzUcPNlK16zSmOvPJyjd0bqUcPUz3mNw1fCg).

**(i) Functional analyses of genes in cluster 1.** The bioinformatics analysis of genes present in cluster 1 (Fig. S5A) showed significant enrichment in 18 GO terms. It highlighted genes encoding proteins related to lipid transport (steroid-binding activity [GO:0005496], fold enrichment [FE] of 15.4, and lipid transporter activity [GO:0005319], FE of 5.7, including oxysterol binding protein and phospholipid-transporting ATPase). It also highlighted genes encoding proteins related to catalytic activity on DNA and RNA (GO:140097, FE of 4.6, and GO:140098, FE of 3.7, including DNA ligase, ATP-dependent RNA helicase, recQ family DNA helicase, tRNA-dihydrouridine synthetase, and tRNA synthetase (Arg, Leu, Ala, Lys, Thr, Ile, Glut, and His). Cluster 1 was also enriched in genes encoding a cytoskeletal binding protein (GO:0008092, FE of 4.1), including kinesin, dynamin, filamin, and villidin, and heat shock protein binding (GO:0031072, FE of 3.4), including heat shock protein 70.

**(ii) Functional analyses of genes in cluster 2.** The bioinformatics analysis of genes present in cluster 2 showed significant enrichment in six GO terms, all related to small GTPases that appeared in several combinations in the GO term enrichment analysis (Fig. S5B). These include nucleoside triphosphate regulator activity (GO:0060589, FE of 3.3), GTPase regulator activity (GO:0030695, FE of 3.45, and GO:005096, FE of 3.49), Ras GTPase binding (GO:0017016, FE of 4.69), small GTPase biding (GO:0031267, FE of 4.69), and Rab GTPase binding (GO:0017137, FE of 5.2).

**(iii) Functional analyses of genes in cluster 3.** The bioinformatics analysis of genes present in cluster 3 did not show significant enrichment.

**(iv) Functional analyses of genes in cluster 4.** The bioinformatics analysis of genes present in cluster 4 showed significant enrichment in 5 GO terms related to ribosomal proteins that appeared in several combinations in the GO term enrichment analysis (Fig. S5C). These include ribosome binding (GO:0043022, FE of 15.6), ribonucleoprotein complex binding (GO:0043021, FE of 9.1), protein-containing complex binding (GO:0044877, FE of 6.09), structural constituent of ribosome (GO:0003735, of FE: 4.0), and structural molecule activity (GO:0005198, FE of 3.38).

### Comparative analysis of genes upregulated in virulent E. histolytica trophozoites versus genes downregulated in trophozoites that were grown with queuine.

We compared our RNA-Seq data containing genes downregulated in trophozoites that were grown with queuine with the previously published RNA-Seq data of genes upregulated in a highly virulent E. histolytica HM1:IMSS strain (36). Our analysis showed that of 816 upregulated genes present in the virulent strain versus 664 genes downregulated in trophozoites that were grown with queuine, a total of 70 genes were common between both sets (see Table S2 available at https://datadryad.org/stash/share/Y6qAAIhEzUcPNlK16zSmOvPJyjd0bqUcPUz3mNw1fCg). These genes include putative cysteine protease (EHI_151440), putative thioredoxin (EHI_152600 and EHI_124400), fatty acid elongase (EHI_009370), ubiquitin putative (EHI_156660), and putative eukaryotic elongation factors (EHI_006170), and a majority of the genes correspond to hypothetical proteins that are shared between the two data sets.

Functional analysis of the proteins in this set showed that the most abundant classes of proteins were protein modifying enzyme (PC00260 and PC00171) (30%), nucleic acid binding protein (PC00171) (20%), translational protein (PC00263) (20%), transporter (PC00227) (10%), protein-binding activity modulator (PC00095) (10%), and metabolite interconversion enzyme (PC00262) (10%) (Table S2).

Taken together, this transcriptome analysis indicates that queuine induces the upregulation of genes involved in OS response and the downregulation of genes previously associated with virulence.

### Codon usage frequency in genes which are upregulated or downregulated in trophozoites cultivated in the presence of queuine.

To gain insights into the role of Q-tRNA modification on translation in E. histolytica, we compared the gene-specific codon usage (GSCU) frequency of each transcript upregulated in the parasite cultivated in the presence of queuine to the average codon usage frequency defined by the entire set of E. histolytica genes (see Table S5A available at https://datadryad.org/stash/share/Y6qAAIhEzUcPNlK16zSmOvPJyjd0bqUcPUz3mNw1fCg). Previously, GSCU analysis has been used to analyze all genes in a genome to identify those overusing specific codons translationally enhanced by wobble U modifications (37), and here, we have advanced the methodology to analyze transcriptionally regulated transcripts (Table S5B). We observed that some groups of genes have a distinct codon bias, but we were not able to link these gene clusters back specifically to codons decoded by Q (GUN anticodon) (see Fig. S6A at the above-listed URL). Most likely, the use of all 62 codons in our analysis obscured the clustering, as strong biases in many codons can be observed for a group of queuine-regulated genes. We next stratified the transcripts upregulated in the parasite cultivated in the presence of queuine based on whether they overuse the four U (U-GUN)- or four C (C-GUN)-ending codons decoded by Q (Table S5C). We observed two distinct groups that were classified according to the protein class which they encode (see Fig. S7 at the URL mentioned above). Of the genes that overuse the C (C-GUN), genes encoding translational protein (PC00263) such as elongation factor 2 (EHI_166810, EHI_189490, and EHI_164510) and numerous ribosomal proteins EHI_093580, EHI_161180, EHI_199990, and EHI_055670) were the most abundant (Fig. S7A). Of the genes that overuse the U (U-GUN), genes encoding proteins associated with nucleic acid binding (PC00171) such as transcription initiation factor IIIB (EHI_158020), CCR4/NOT transcription complex subunit 3 (EHI_119550), or the zinc finger protein (EHI_055640) were the most abundant (Fig. S7B). They are followed by genes encoding metabolite interconversion enzyme (PC00262) such as NAD FAD-dependent dehydrogenase (EHI_099700), peroxiredoxin (EHI_001420), and NADP-dependent alcohol dehydrogenase (EHI_023110) (Fig. S7B).

## DISCUSSION

We have recently demonstrated that E. histolytica uses oxaloacetate produced by E. coli to protect itself against OS (9). In this study, we present another facet of this protective effect through the lens of the human microbiome. Queuine released by the gut bacteria has a protective effect on the parasite during OS. Interestingly, this protective effect against OS conferred by queuine is also found in cancer cells, where the activity of antioxidant enzymes is improved by the supplementation of queuine in the culture (20), suggesting that this effect is universal.

In this work, we have shown for the first time that E. histolytica has an active TGT enzyme. EhTGT activity is essential for the incorporation of queuine in tRNAs and the resistance to OS. EhTGT shares structural similarity with its eukaryotic counterpart which is also a heterodimer. It has been proposed that one subunit (QTRT1) is responsible for the recognition of the anticodon loop of the tRNA and transglycosylase activity, whereas the second subunit (QTRTD1) is responsible for maintaining the orientation of the tRNA (38, 39). It has been suggested that the human QTRTD1 salvages queuine from queuosine-5′-monophosphate that is generated after tRNA turnover (40). Since the amount of queuine which circulates is in the range of 1 to 10 nM (24), it might be possible that in E. histolytica, there is a requirement for a second subunit to salvage Q from microbial tRNAs. Alternatively, the parasite may rely on a dedicated enzymatic machinery to salvage Q. DUF2419 is a protein with structural similarity to DNA glycosidases that has been involved in Q salvage in S. pombe (41). E. histolytica expresses a gene, EHI_098190, which is strongly homologous to S. pombe DUF2419 (query cover, 97%; E value, 1E−28; percentage identity, 27.1%). Work is in progress to characterize the involvement of EhDUF2419 in the salvage of Q from bacteria.

Different mechanisms can explain why queuine protects E. histolytica against OS. The first mechanism described in S. pombe and mammals involves the stimulation of Dnmt2 activity by prior queuosine incorporation at G34 of ${\text{tRNA}}_{\text{GUC}}^{\text{Asp}}$ (23, 42). Dnmt2-mediated C38 methylation of ${\text{tRNA}}_{\text{GUC}}^{\text{Asp}}$ during OS protects it against cleavage by the RNA endonuclease angiogenin (43, 44). In E. histolytica, we have also correlated the hypermethylation of ${\text{tRNA}}_{\text{GUC}}^{\text{Asp}}$ catalyzed by the E. histolytica Dnmt2 homolog Ehmeth to the resistance of the parasite to OS (18). Then, this specific ${\text{tRNA}}_{\text{GUC}}^{\text{Asp}}$ is preferentially used by the cells for the translation of stress proteins (26). In our present study, we have shown that exogenous supplementation of E. histolytica trophozoites with queuine leads to hypermethylation of C38 in ${\text{tRNA}}_{\text{GUC}}^{\text{Asp}}$ and that this hypermethylation depends on the active EhTGT enzyme. Thus, by extrapolating this information, it is possible that queuine exerts a protective effect on E. histolytica during OS via the hypermethylation of C38 in ${\text{tRNA}}_{\text{GUC}}^{\text{Asp}}$. The fact that EhTGT has a cytoplasmic localization (this work) whereas Ehmeth has a nuclear localization (45) does not rule out this possibility, because tRNAs are able to shuttle between the cytoplasm and the nucleus (46).

In S. pombe, Q-tRNA modification enhances the translational speed of the C-ending codons for Asp (GAC) and histidine (CAC) and reduces that of U-ending codons for asparagine (AAU) and tyrosine (UAU) (21). In human cells, Q-tRNA modification also has an effect on translational speed, but contrary to S. pombe, it increases translational speed of the U-ending codons (17). Although we did not determine in this study the effect of Q-tRNA modification on the translational speed of C- versus U-ending codons, we were able to identify among the genes upregulated in the parasite cultivated in the presence of queuine two groups that either overuse the four U (U-GUN)- or the four C (C-GUN)-ending codons decoded by Q. Remarkably, the most abundant group that overuses the U (U-GUN)-ending codons includes possible transcription factors and proteins involved in OS resistance (47–49). In contrast, the most abundant group that overuses the C (C-GUN)-ending codons includes proteins involved in translation. In regards to the effect that queuine has on regulating gene expression, increasing resistance to OS, and reducing translation in E. histolytica, it is tempting to speculate that U (U-GUN)-ending codons will be associated with preferentially translated proteins, whereas C (C-GUN)-ending codons will be associated with proteins with reduced translation. Further translational, RNA modification, and codon analytic studies will be used in the future to test these predictions.

The second mechanism of protection against OS mediated by queuine relies on its ability to prepare the parasite to resist OS. We have reported here that several genes involved in stress response are upregulated in the presence of queuine, including heat shock protein 70 (Hsp70), antioxidant enzymes, and enzymes involved in DNA repair. Hsp70 is known for its role in the refolding of denatured and misfolded proteins and translocation of secretory proteins (50). In E. histolytica, Hsp70 is produced by cells in response to OS and in the heat shock response, and its levels are also increased during the formation of liver abscess (51–53). Antioxidant enzymes such as alcohol dehydrogenases (ADH2) are well known for their role in energy metabolism in E. histolytica during OS (54) and help the parasite to resist OS (55, 56). Transketolase is another antioxidant enzyme that is involved in the pentose phosphate pathway. This enzyme is essential for the production of the antioxidant NADPH, which ultimately protects cancer cells against OS (57, 58). The upregulation of the expression of this enzyme in the parasite exposed to queuine (this work) and the accumulation of sedoheptulose-7-phosphate, the product of transketolase, in the parasite exposed to OS (59) suggest that this enzyme is also involved in the resistance of the parasite to OS. RecQ helicases are conserved across all forms of life, ranging from prokaryotes to eukaryotes (60). They have many metabolic functions in the cell but are widely popular for their role in DNA repair and genome stability (61–63). Mutations in these enzymes lead to chromosomal instability, which in turn is associated with many diseases such as cancer (64). Human RecQ helicase is involved in the repair of oxidative DNA damage (64, 65), and it interacts with poly(ADP-ribose) polymerase 1 (PARP-1) both *in vivo* and *in vitro*, aiding in the repair (66). This interaction with PARP-1 is interesting, as PARP-1 itself is known for its involvement in DNA repair and also in the regulation of transcription in the cell (67). Based on these observations, we hypothesize that either queuine stimulates the production of DNA-repairing enzymes in E. histolytica under normal physiological conditions or it prepares the parasite to resist DNA damage in response to stress. These results suggest that queuine has a role in DNA repair damage in the parasite; however, this assumption needs thorough investigation.

In this work, we have shown that queuine impairs the cytopathic activity of the parasite and its survival in the large intestine of mice with experimentally induced amebiasis. The transcriptome of the parasite exposed to queuine provides some insights into the mechanism that leads to this low-virulence phenotype. Many protein-modifying enzymes such as cysteine proteases, metalloproteases, phosphatases, and ubiquitin ligases have their expression downregulated in the presence of queuine. Protein phosphatases have an essential role in cellular signaling pathways, and they may also play a role in the pathogenicity of the parasite (68). GTPases are an essential part of cell signaling events, and we found many genes belonging to putative Ras GTPases, Rho GTPase, and Rab GTPase proteins. Many of these genes have been linked to vesicular trafficking. For example, Rab GTPases may play an essential role in phagocytosis (69). Moreover, a Rab GTPase in E. histolytica (EhRab7A) is involved in the transport of cysteine protease to phagosomes (70). Further investigation regarding these enzymes will help us define their roles in the virulence of the parasite. The most surprising observation in genes downregulated in response to queuine is the presence of 760 genes encoding hypothetical proteins. These proteins whose functions are unknown probably play important roles in regulating the virulence of the parasite.

Another explanation as to how or why queuine may affect the virulence of the parasite stems from our comparative analysis of genes upregulated in the virulent strain (36) that are downregulated in the presence of queuine. For example, actin, which is a major cytoskeletal protein involved in motility of the parasite and phagocytosis (71), has its expression upregulated in the virulent strain of E. histolytica and downregulated in the parasite exposed to queuine. The same trend is observed for cysteine proteases, which are essential virulence factors in the parasite that promote intestinal invasion by degrading the extracellular matrix (72). Among the 70 common genes, more than half correspond to hypothetical proteins. Characterization of these genes presents an opportunity to discover their novel functions in the parasite. Moreover, new hypothetical proteins may serve as good markers and act as possible pharmacological targets against this parasite.

Our results may provide some clues to understand why most of the infections with E. histolytica are asymptomatic. Factors leading to such an outcome are the level of virulence of the parasite, the immune status of the host, and most probably the gut microbiota. Different mechanisms may influence the development of amoebiasis by the gut microbiota, including competition with the parasite to invade the colonic mucosa or the boosting of a host immune response against the parasite (73, 74). Here, we are adding a new mechanism to this list that is based on the downregulation of virulence genes expression by queuine. The fact that queuine confers resistance to OS seems counterintuitive, because this resistance often correlates with a stronger virulence of the parasite (48, 75). However, the resistance to OS conferred by queuine is mild if we compare it to the strong resistance conferred by oxaloacetate, an antioxidant metabolite scavenged by the parasite from the gut microbiota (9). This mild resistance to OS may be enough to help the parasite survive fluctuation of the oxygen content that occurs in the large intestine due to changes in the composition of the microbiota. For example, the depletion of butyrate-producing species following an antibiotic treatment increased epithelial oxygenation and allows the expansion of aerobic luminal microbes (76). The price paid by the parasite to acquire a mild resistance to OS is a lower virulence. The concept of fitness cost is well known and it has been described for many microorganisms (77) that have acquired drug resistance, including E. histolytica (78).

In this work, we have shown that queuine improves protein translation in control trophozoites and in trophozoites exposed to OS. Queuine can act at the level of translational speed, translational accuracy, protection of Q-tRNA against cleavage, and aminoacylation (42). Our attempts to separate aminoacylated from nonaminoacylated ${\text{tRNA}}_{\text{GUC}}^{\text{Asp}}$ or ${\text{tRNA}}_{\text{GUG}}^{\text{His}}$ by using acid urea polyacrylamide gel (79) were not successful. Consequently, we could not test the effect that queuine has on tRNA aminoacylation. In E. histolytica and in other systems (80), stress is partially regulated by the phosphorylation of the alpha subunit of eukaryotic initiation factor 2 (eIF2) that leads to the inhibition of eIF2α activity, resulting in a general decline in protein synthesis. It is possible that queuine reduces the phosphorylation level of eIF2α and consequently improves protein synthesis. Work is in progress to test this hypothesis.

Queuine regulates the transcription of more than 1,200 genes. A different hypothesis can explain the effect of Q on the transcriptome of E. histolytica: the presence of queuosine in the anticodon loop position 34 of ${\text{tRNA}}_{\text{GUC}}^{\text{Asp}}$ and maybe other tRNAs influences the transcriptome of E. histolytica.

This hypothesis is supported by a recent report showing that the deletion of a gene encoding KEOPS (an enzyme involved in the catalysis of t6A modification in tRNA) in Saccharomyces cerevisiae leads to the upregulation of the transcription of genes related to mitochondrial function and carbohydrate metabolism (81). The effect of tRNA modifications on transcription can also be indirect when it impairs the translation Gcn4, a transcription factor (81). It is possible that, like Gcn4, a yet-to-be discovered transcription factor whose translation depends on the presence of Q-tRNAs is involved in the regulation of gene expression in Q trophozoites. It is also possible that Q is regulating transcription by using a mechanism that does not involve the modification of tRNA. An alternative explanation is that queuine regulates the expression of a transcription factor that controls the expression of these genes. The best-studied example is in the enteropathogenic bacteria, Shigella flexneri. VirF has been recognized as the main transcriptional regulator involved in the virulence of this pathogen. Lack of this VirF mRNA (in the bacteria lacking TGT) reduces the virulence of the bacteria. It has been found that VirF mRNA is a substrate of the E. coli TGT, since it has extremely high sequence similarity with *Shigella* TGT, and that this recognition causes a site-specific modification of a base in the VirF mRNA and modulates its translation (34). Based on this information, we cannot rule out that EhTGT and queuine work via a mechanism unrelated to tRNA modification.

To conclude, we have investigated for the first time the role of queuine, a compound produced by the gut microbiota, on the physiology of E. histolytica. We showed that queuine is incorporated in the tRNA of the parasite and that it prepares the amoeba to resist oxidative stress *in vitro.* We have also shown that the presence of queuine leads to hypermethylation of ${\text{tRNA}}_{\text{GUC}}^{\text{Asp}}$ in the organism, and this agrees with other studies indicating that the role of queuine in methylation is conserved across all species and that its presence may influence Dnmt2 activity. Our study also revealed the role of queuine in shaping the transcriptome of the parasite. Our data also support the role of queuine in the resistance of the parasite to OS, but this comes at a cost in terms of virulence. Antivirulence therapeutic strategies have been proposed to fight against drug-resistant pathogens (82). In this regard, queuine provided directly to the patient or by selected probiotics may represent a new therapeutic approach for the treatment of amoebiasis.

## MATERIALS AND METHODS

### E. histolytica culture.

E. histolytica trophozoites, HM-1:IMSS strain, were grown under axenic condition at 37°C in TYI-S-33 medium prepared according to a previously reported protocol (83). The trophozoites were harvested during the logarithmic phase of growth by chilling the culture tubes at 4°C and pelleted by centrifugation at 500 × *g* for 5 min. The pellet was washed twice with ice-cold phosphate-buffered saline. Trophozoites were grown with 0.1 μM queuine (a kind gift from Klaus Reuter, University of Marburg) for 3 days.

### Bacterial strains.

The bacterial strains used in this study are E. coli BL21(DE3)pLysS and DH5 alpha. E. coli was grown at 37°C in Luria-Bertani (LB) medium (84).

### Cultivation of HeLa cells.

HeLa cells (a kind gift from T. Kleinberger, Faculty of Medicine, Technion) were maintained in continuous culture using a previously described protocol (85).

### Transfection of E. histolytica trophozoites.

The transfection of E. histolytica trophozoites was performed using a previously described protocol (86).

### Effect of queuine on hydrogen peroxide resistance.

E. histolytica trophozoites were grown with and without queuine (0.1 μM) for 3 days at 37°C. Trophozoites were harvested (1 × 10^6^) and exposed in TYI-S-33 medium (without serum) to various concentrations of H_2_O_2_ (0 to 5 mM) for 30 min at 37°C. The viability of the trophozoites was determined with the eosin dye exclusion method (9), and this value was used to calculate the median lethal dose (LD_50_) of H_2_O_2_.

### Measurement of the cytopathic activity of E. histolytica.

The cytopathic activity of E. histolytica trophozoites was determined using a previously described protocol (87). Briefly, E. histolytica trophozoites (2.5 × 10^5^ cells/per well) that were grown with and without queuine were exposed or not to 2.5 mM H_2_O_2_ for 30 min in TYI-S-33 medium (without serum). After the incubation, the cells were resuspended in fresh TYI-S-33 medium (without serum) before their incubation with HeLa cell monolayers in 24-well tissue culture plates at 37°C for 40 min or 90 min. The incubation was stopped by placing the plates on ice, and unattached trophozoites were removed by washing the plates with cold phosphate-buffered saline (PBS) supplemented with 1% galactose. The cells were fixed with 4% formaldehyde for 10 min and finally washed twice with cold PBS. HeLa cells remaining attached to the plates were stained with methylene blue (0.1% in 0.1 M borate buffer, pH 8.7). The dye was extracted from the stained cells by 0.1 M HCl, and its color intensity was measured spectrophotometrically as an optical density at 660 nm (OD_660_). The amount of dye extracted was proportional to the number of intact cells that remained attached to the tissue culture well. The amount of dye extracted from monolayers of tissue cultured cells that had not interacted with trophozoites served as a control (100% of monolayer integrity). The intensity of color was measured in a spectrophotometer (at 660 nm) after appropriate dilution with HCl (0.1 M). Experiments were carried out in duplicates, repeated at least twice, and standard error was determined. To decide the exact time at which to terminate an experiment, the progress in tissue culture damage during the incubation with trophozoites was also monitored by periodic observation under an inverted microscope.

### Primers used in this study.

The primers used in this study are listed in Table 4.

**TABLE 4:** List of all the primers used in this study

Primer name	Primer sequence^*a*^	Direction
EhQTRT1 5′	ATGAGTGCTCTTTCATTGCG	Sense
EhQTRT1 3′	TGCAAACTCTACACCAACT	Antisense
5′ EhQTRT1 BglII	AGATCTGATGAGTGCTCTTTCATTG	Sense
NotI EhQTRT1 3′	GCGGCCGCTGCAAACTCTACACCAACT	Antisense
EhQTRTD1 BglII 5′	AGATCTCATGTCAACTTCAACACCACA	Sense
EhQTRTD1 KpnI 3′	GGTACCTCAATCATCATCCTCATTT	Antisense
${\text{tRNA}}_{\text{GUC}}^{\text{Asp}}$ stem & loop 3′	CTCAACTGGATTGGCTCTCTTCAACGGGGATTC	Antisense
${\text{tRNA}}_{\text{GUC}}^{\text{Asp}}$ 5′	ATAGTATAGTTGGTTAGTATT	Sense
Universal primer 5′ ${\text{tRNA}}_{\text{GUC}}^{\text{Asp}}$	CGCGCGAAGCTTAATACGACTCACTATACTCTTCAATAGTATAGC	Sense
${\text{tRNA}}_{\text{GUC}}^{\text{Asp}}$ 3′	TGGTCTTCAACGGGGATTCGAAC	Antisense
BamHI EhQTRT1 5′	GGATCCGATGAGTGCTCTTTCATTGCG	Sense
1-265-EhQTRT1-XhoI 3′	ATATCTCGAGATCAACTCCAAATAATAC	Antisense
266-EhQTRT1-Xho 5′	ATATCTCGAGATGTTTGCTTGUGTTTAT	Sense
tRNA^His^_GUG_ 5′ biotinylated	TGCTCACACCAGGAATCGAACCTGGGTT	Sense
${\text{tRNA}}_{\text{GUC}}^{\text{Asp}}$ 5′ biotinylated	CTCTCCAACGGGGATTCGAACCCCGGTCTTTC	
18s rRNA EntaF	ATGCACGAGAGCGAAAGCAT	Sense
18s rRNA EhR	GATCTAGAAACAATGCTTCTCT	Antisense

a Restriction sites are underlined.

### Ethics statement.

Animal procedures dealing with the mouse model of intestinal amebiasis were approved by the Institutional Animal Care and Use Committee (number [no.] 211075) and conducted at the AAALAC-accredited National Institute of Infectious Diseases, Japan. Animal procedures dealing with the production of EhTGT antibody were approved by the Technion Animal Care and Use Committee (IL-115-10-16) and conducted at the accredited Faculty of Medicine-Technion, Haifa, Israel.

### Detection and quantification of E. histolytica in infected mouse stool.

The virulent trophozoites were passaged *in vivo* through the ceca of CBA/J mice (purchased from Jackson Laboratory, Japan) (88). For all intracecal injections, 1 × 10^6^ axenic trophozoites with or without 0.1 μM queuine treatment for 3 days were inoculated into the proximal and apical regions of the cecum (89). To quantify the number of E. histolytica trophozoites in stool, real-time quantitative PCR was performed by using Fast SYBR green master mix (Thermo Fisher Scientific, USA) in the StepOne Plus real-time PCR system (Applied Biosystems, USA). Primer sets were specific to E. histolytica 18S rRNA (EntaF and EhR) (9, 90). A standard curve was generated using the DNA extracted from trophozoites serially diluted from 10^5^ to 10^0^. Using the standard curve and the stool weight, the number of trophozoites per milligram stool was calculated.

### Construction of the pETDuet-1 His-tagged EhQTRT1-EhQTRTD1 vector.

For the construction of the pETDuet-1 His-tagged EhQTRT1-EhQTRTD1 vector, EhQTRT1 was amplified from E. histolytica genomic DNA with the primers BamHI EhQTRT1 and NotI EhQTRT1. The PCR product was cloned into a pGEM-T easy vector, digested with BamHI and NotI, and then subcloned in a pDuet expression vector that was previously linearized with BamHI and NotI to generate the pETDuet-1 His-tagged EhQTRT1 vector. EhQTRTD1 (EHI_079900) was then amplified from E. histolytica cDNA by using primers BglII EhQTRTD1 and KpnI EhQTRTD1 and then cloned into the pGEM-T easy vector, digested with BglII and KpnI, and subcloned in the pETDuet-1 His-tagged EhQTRT1 vector that was previously linearized with BglII and KpnI.

### Construction of the pETDuet-1 his-tagged mEhQTRT1-EhQTRTD1 vector.

For the construction of the pETDuet-1 his-tagged mEhQTRT1-EhQTRTD1 having a point mutation of D267 to A267 in EhQTRT1, EhQTRT1 was amplified from E. histolytica genomic DNA using primers standard EhQTRT1 and 1-265-EhQTRT1 XhoI. The second part of EhQTRT1 was amplified using genomic DNA using primers 266-EhQTRT-XhoI (where the aspartate was replaced with alanine in the primer) and reverse primer EhQTRT1. Both PCR products were purified using a QIAquick PCR purification kit (Qiagen), digested with XhoI for 1 h at 37°C, and repurified. The purified products were ligated at 16°C overnight using T4 ligase. Following ligation, the product was amplified by PCR using primers BamHI EhQTRT1 and NotI EhQTRT1. The PCR product was cloned in to the pGEM-T easy vector, digested with BamHI and NotI, and further subcloned into a pETDuet-1 vector that was previously linearized with BamHI and NotI. Following this, the EhQTRTD1 gene was cloned into the pETDuet-1 vector as described above. The resulting plasmid was sequenced to ensure the presence of the desired mutation.

### Construction of silenced EhQTRT1 vector.

For the construction of the siEhQTRT1 vector, the entire protein-coding region of EhQTRT1 was amplified from E. histolytica genomic DNA with 5′ EhQTRT1 BglII and 3′ EhQTRT1 XhoI primers. The resulting PCR product was cloned into the pGEM-T easy vector system (Promega), digested with BglII and XhoI, and then subcloned into a pEhEx-04-trigger vector containing a 142-bp trigger region (EHI_048660) (a kind gift of Tomoyoshi Nozaki, University of Tokyo, Japan) to generate siEhQTRT1.

Plasmids were sequenced to ensure the presence of unwanted mutations.

### Growth rate assays.

Trophozoites (3 × 10^3^/ml) were grown in a 13-ml tube in TYI-S-33 medium at 37°C, and the number of viable trophozoites was counted at 24, 48, and 72 h by using the eosin dye exclusion method.

### Preparation of recombinant EhTGT complex.

A recombinant EhTGT complex was expressed as His-tagged protein in E. coli BL21(DE3)pLysS competent cells, which were transfected with the pETDuet-1 vector-derived plasmids. The overnight culture was supplemented with ampicillin (100 μg/ml) and grown at 37°C. The culture was grown with the addition of 100 μM ZnSO_4_ until the OD_600_ reached 0.6. The synthesis of the His-tagged protein complex was initiated by adding isopropyl β-d-1-thiogalactopyranoside (IPTG) to the culture at a final concentration of 1 mM. After overnight incubation in the presence of IPTG at 22°C, the bacteria were harvested and lysed and the His-tagged proteins were purified with nickel-nitrilotriacetic acid (Ni-NTA) agarose beads (Protino; Macherey-Nagel) as described elsewhere (85). The proteins were then eluted from the Ni-NTA agarose beads with elution buffer (50 mM NaH_2_PO_4_ [pH 8.0], 300 mM NaCl, and 250 mM imidazole).

The eluents were further analyzed by SDS-PAGE, the fractions containing EhQTRT1-EhQTRTD1 were combined, and then dialysis was performed in dialysis spin columns with a dialysis buffer (25 mM HEPES [pH 7.4], 300 mM NaCl, 2 mM dithiothreitol [DTT]). Two rounds of dialysis were performed for 4 h each, one at room temperature and the second round of dialysis at 4°C. The proteins were combined, concentrated, and then applied to a Superdex 200 increase 10/300 GL column for size exclusion chromatography. Following their elution from the column with the elution buffer (25 mM HEPES [pH 7.3], 100 mM NaCl, 50 μM ZnSO_4_, 5% glycerol, and 2 mM DTT), the eluted fractions (corresponding to the peak) were analyzed by SDS-PAGE, followed by silver staining. The same procedure was used for the purification of mEhQTRT1-QTRTD1.

### Preparation and purification of ${\text{tRNA}}_{\text{GUC}}^{\text{Asp}}$ and ${\text{tRNA}}_{\text{CAC}}^{\text{Val}}$.

A commercially synthesized DNA oligomer containing the ${\text{tRNA}}_{\text{GUC}}^{\text{Asp}}$ sequence of E. histolytica and the T7 promoter sequence was amplified by using PCR using universal primer 5′ ${\text{tRNA}}_{\text{GUC}}^{\text{Asp}}$ and ${\text{tRNA}}_{\text{GUC}}^{\text{Asp}}$ 3′ under with the following: primers (20 pmol each), ${\text{tRNA}}_{\text{GUC}}^{\text{Asp}}$ template (500 ng), Mg^2+^ (2 mM), deoxynucleoside triphosphates (dNTPs; 0.5 mM each), ThermolPol *Taq* buffer (1×), *Taq* DNA polymerase (2 U), in a final volume of 50 μl. The samples were treated at 94°C (5 min), followed by 29 cycles of 94°C (45 s), 60°C (45 s), and 72°C (45 s). A final extension reaction was allowed to occur at 72°C for 10 min. The PCR product was purified by using the QIAquick PCR purification kit (Qiagen). ${\text{tRNA}}_{\text{GUC}}^{\text{Asp}}$ was subsequently generated by *in vitro* transcription. The 50-μl reaction mixtures contained the following: T7 RNA Pol buffer (10×), 5 μl; ribonucleoside triphosphates (rNTPs; 25 mM each), 4 μl; template, 300 to 400 ng; T7 RNA polymerase, 4 μl; and purified water up to the final volume. The reaction mixture was incubated overnight at 37°C, after which, white precipitates (magnesium pyrophosphate) were removed by centrifugation (13,000 rpm, 5 min). DNase I was added to remove any unwanted DNA templates in the reaction mixture and incubated for another 30 min. The tRNA transcript was ethanol precipitated at −20°C and then pelleted by centrifugation (20,000 × *g*, 30 min, 4°C). The resulting pellet was resuspended in diethyl pyrocarbonate (DEPC)-treated water, and the concentration was measured using a NanoDrop (Thermo Scientific). ${\text{tRNA}}_{\text{CAC}}^{\text{Val}}$ (EHI_034860) was synthesized as an ultramer desalted RNA oligonucleotide (86.2 μg total) by Syntezza Bioscience (Israel).

### Enzymatic assay with [^14^C]guanine.

The enzyme activity of the EhTGT complex was performed as described elsewhere with some modifications using 8-[^14^C]guanine to quantify activity by scintillation counting (40). Briefly, the kinetic assays were set up with 3 μg of *in vitro* transcribed ${\text{tRNA}}_{\text{GUC}}^{\text{Asp}}$ or with 3 μg of synthetic ${\text{tRNA}}_{\text{CAC}}^{\text{Val}}$, 0.5 μl of 8-[^14^C]guanine (57 mCi/mmol), 0 to 15 μg of EhTGT enzyme freshly purified by Ni-NTA chromatography, and HEPES reaction buffer (100 mM HEPES [pH 7.3], 20 mM MgCl_2_, and 5 mM DTT) to a final volume of 50 μl. Studies were performed in triplicates, and samples were incubated at 37°C. Aliquots were taken every 30 min for a period of 2 h and quenched in 3 ml of 5% trichloroacetic acid (TCA) on Whatman-glass fiber filters (GE Healthcare) and washed 3 times with 1 ml ethanol to dry the filter. The filters were then added to a scintillation vial containing 3 ml of scintillation fluid, and the samples were analyzed in a scintillation counter where counts were reported as counts per minute.

### Production of EhTGT antibody.

Female BALB/c mice were injected intraperitoneally with 700 μg recombinant His-tagged TGT protein that was emulsified in complete Freund’s adjuvant (Sigma-Aldrich). Two weeks later, the mice were injected with 700 μg of His-tagged TGT complex protein in incomplete Freund’s adjuvant (Sigma-Aldrich). One week after the 6-week injection, the mice were anesthetized, and their blood was collected as described elsewhere (85). The serum that was obtained from mice that were not injected with His-tagged EhTGT complex was used as the control.

### Western blotting.

Total protein extracts of E. histolytica trophozoites were prepared according to a published method (91). Proteins (40 μg) in the total extract were resolved on a 10% SDS-PAGE gel in SDS-PAGE running buffer (25 mM Tris, 192 mM glycine, 0.1% SDS). The resultant protein bands were visualized after staining with Ponceau red stain. Next, proteins were electrotransferred in protein transfer buffer (25 mM Tris, 192 mM glycine, 20% methanol, pH 8.3) to nitrocellulose membranes (Whatman, Protran BA83). The blots were first blocked using 3% skim milk and then probed with 1:1,000 mouse polyclonal EhTGT (complex) antibody or 1:1,000 mouse monoclonal actin antibody, clone c-4 (MP Biotechnologies, CA, USA) for 16 h at 4°C. After incubation with one of the previously described primary antibodies, the blots were incubated with 1:5,000 secondary antibody (Jackson ImmunoResearch) for 1 h at room temperature (RT), and then developed using enhanced chemiluminescence (Bio-Rad).

### SUnSET assay to measure protein synthesis.

Trophozoites (2 × 10^6^/ml) were grown with and without queuine (0.1 μM for 3 days at 37°C) and were exposed or not to 2.5 mM H_2_O_2_ for 20 min at 37°C. After treatment with H_2_O_2_, the trophozoites were incubated with 10 μg/ml puromycin (Sigma) for 20 min. For pretreatment of the trophozoites with cycloheximide (Sigma), the trophozoites were incubated with 100 μg/ml cycloheximide for 5 min before the addition of puromycin. The trophozoites were lysed with 1% IGEPAL (Sigma) in phosphate-buffered saline (PBS) with protease inhibitors. Puromycin was detected by immunoblotting as described above with a 12D10 clone monoclonal puromycin antibody (Millipore). The amount of total protein in each lane was determined by using the No-Stain protein labeling reagent (Thermo Fisher Scientific). Imaging for puromycin and total protein signals was performed on a FUSION FX7 EDGE imaging system (Witec AG). Quantification of signal density was performed using ImageJ.

### RNA extraction.

Total RNA was extracted from control trophozoites, trophozoites treated with queuine (0.1 μM for 3 days at 37°C), and those exposed (or not) to H_2_O_2_ (in three biological replicates) using the TRI reagent kit according to the manufacturer’s instructions (Sigma-Aldrich). Libraries were built using a TruSeq mRNA-Seq library preparation kit (Illumina), according to the manufacturer’s recommendations. Quality control was performed on an Agilent Bioanalyzer. Sequencing was performed on a HiSeq 2500 system (Illumina) and produced 65-base single-end reads.

### RNA-Seq data analysis.

Bioinformatics analysis was performed using the RNA-Seq pipeline from Sequana (92). Reads were cleaned of adapter sequences and low-quality sequences using cutadapt version 1.11 (93). Only sequences at least 25 nucleotides (nt) in length were considered for further analysis. Bowtie version 1.2.2 (94), with default parameters, was used for alignment on the reference genome (Entamoeba histolytica, from amoebaDB). Genes were counted using featureCounts version 1.4.6-p3 (95) from the Subreads package (parameters: -t gene -g ID -s 1).

Count data were then analyzed using R version 3.5.3 (12) and the Bioconductor package DESeq2 version 1.22.2 (30). Normalization and dispersion estimation were performed with DESeq2 (using the default parameters), and statistical tests for differential expression were performed applying the independent filtering algorithm. A generalized linear model including (i) the effect of stress (+OS versus −OS), (ii) the presence of queuine (Q versus T), and (iii) the interaction term was set up to test for intercondition differences in expression and to test the interaction between stress and queuine. For each pairwise comparison, raw *P* values were adjusted for multiple testing using the Benjamini and Hochberg procedure (96). Genes with an adjusted *P* value of <0.05 and a fold change of >2 were considered to be differentially expressed.

Principal-component analysis and hierarchical clustering were performed using variance-stabilizing transformed counts. Hierarchical clustering of the genes was based on the correlation distance and the Ward aggregation criterion. The average profile was established by computing the mean log_2_-normalized count over the genes in each of the four identified clusters.

The functional classification, GO term analysis, and protein class analysis were performed with PANTHER tools (http://pantherdb.org) (97).

To determine the gene-specific codon usage (GSCU) for each transcript upregulated in trophozoites cultivated in the presence of queuine, we obtained the protein-coding sequences for 7,930 E. histolytica genes from the Amoeba database (https://amoebadb.org/amoeba/app/) The E. histolytica open reading frames were each analyzed using GSCU scripts and methodology previously reported (98). In brief, GSCU analysis determined the total number of times each codon was used in each gene. GSCU analysis also determined the frequency that each codon was used in each gene, with the codon frequency relative to that of the other synonymous codons specific to each individual amino acid. For example, the summed frequency for the two glutamic acid codons (GAA and GAG) potentially used in a gene equal 1.0. Note that methionine and tryptophan codons are omitted from GSCU analysis, as they each only have 1 specifying codon and the GSCU frequency is always 1.0 for AUG (methionine) and 1.0 for UGG (tryptophan). Whether a gene was over- or underrepresented with a specific codon from a synonymous set, relative to the genome average, was determined by calculating a codon specific Z-score, as described previously (98). Gene-specific codon usage data (Z-scores) was analyzed by hierarchical clustering using Cluster 3.0 and visualized as a heat map using Treeview. A spreadsheet containing the gene-specific codon usage data is presented in Table S5 available at https://datadryad.org/stash/share/Y6qAAIhEzUcPNlK16zSmOvPJyjd0bqUcPUz3mNw1fCg.

### Immunofluorescence microscopy.

E. histolytica trophozoites were transferred to microscope slides as described elsewhere (87). The attached trophozoites were washed in warm (37°C) PBS, fixed with methanol, and then permeabilized with fluorescence activated cell sorting (FACS) buffer (2% bovine serum albumin in PBS that was supplemented with 0.1% Tween) as described in reference 87. The slides were then blocked with 5% goat serum in FACS buffer for 15 min at ambient temperature. The samples were then probed with 1:1,000 polyclonal mouse EhTGT (complex) antibody. At the end of the incubation, the slides were washed 3 times in FACS buffer and then incubated with a 1:250 Alexa Fluor antibody (Jackson ImmunoResearch) for 3 h at 4°C. At the end of the incubation, the nuclei of the trophozoites were stained with 1:1,000 4′,6-diamidino-2-phenylindole (DAPI) (Sigma-Aldrich). The samples were then washed with FACS buffer and mounted onto microscope slides with mounting medium (Dako). The specimens were then examined under a confocal immunofluorescence microscope (Zeiss LSM700 meta laser scanning confocal imaging system) with a 63× oil immersion lens objective. For densitometry analysis, the intensity of 15 trophozoites per condition was quantified using ImageJ, and signal intensities were normalized to those of the wild-type trophozoites.

### Northern blot analysis.

Total RNA (10 μg) was suspended in RNA sample buffer comprising 17.5 μl of RNA mix (10 ml RNA mix contains 600 μl of 10× morpholinepropanesulfonic acid [MOPS], 2.1 ml formaldehyde, 6 ml formamide, 120 μg/ml ethidium bromide) and 2 μl of RNA loading buffer. Samples were gently mixed and incubated at 70°C for 5 min and then cooled immediately on ice and loaded into 1% agarose gel (6.66% formaldehyde in 1× MOPS buffer). The RNA was blotted onto a nylon membrane (Amersham Hybond-N) overnight and the membrane was washed briefly in 1× MOPS and cross-linked by UV using 120 mJ (Stratagene UV linker). Membranes were hybridized overnight with ^32^P-labeled EhQTRT1 or enolase probes. The membrane was washed with two washing buffers (washing buffer 1, 40 mM NaP, 1 mM EDTA, 5% SDS; washing buffer 2, 40 mM NaP, 1 mM EDTA, 1% SDS). Images were developed using a phosphorimager, and quantification of signal density from two independent experiments was performed using ImageJ.

### Detection of queuosine in tRNA by chromatography-coupled tandem quadrupole mass spectrometry.

E. histolytica trophozoites were grown with and without queuine (0.1 μM for 3 days), and total RNA was isolated using the TRI reagent kit according to the manufacturer’s instructions (Sigma-Aldrich). Three biological replicates from each treatment condition were processed. Samples were processed as blind samples. tRNA was then purified by size exclusion high-performance liquid chromatography (HPLC) performed using a Bio SEC-3 300 Å (7.8-mm inner diameter by 300-mm length) with 100 mM ammonium acetate (pH 7.4) as the mobile phase. The purity of isolated tRNA species was assessed on a Bioanalyzer 2100 system. The average yield of purified tRNA collected ranged from 0.6 to 0.8 mg per sample. Purified tRNA (5 μg) from each sample was hydrolyzed in a digestion cocktail containing 10 U Benzonase, 5 U bacterial alkaline phosphatases, 0.05 U phosphodiesterase I, 50 μM desferrioxamine, 0.5 μg pentostatin, 50 μM butylated hydroxytoluene, 0.25 μg tetrahydrouridine, and 2.5 nmol of the internal standard [^15^N]_5_dA in 10 mM Tris buffer, pH 7.9. The digestion mixture was incubated at 37°C for 3 h. Digestion mixtures were subsequently removed by passing the mixture through a 10-kDa molecular weight cutoff (MWCO) spin filter. Samples were lyophilized, reconstituted at a final concentration of 100 ng/μl, and analyzed by LC-MS/MS. The ribonucleosides were resolved on a Thermo Scientific Hypersil aQ GOLD column (2.1 mm by 100 mm; 1.9-μm particle size) using a mobile phase gradient consisting of 0.1% formic acid as solvent A and acetonitrile with 0.1% formic acid as solvent B at flow rate of 0.3 ml/min and 25°C using the following elution gradient for solvent B: 0 to 12 min, 0%;12 to 15.3 min, 0 to 1%; 15.3 to 18.7 min, 1 to 6%;18.7 to 20 min, 6%; 20 to 24 min, 6 to 100%; 24 to 27.3 74 min, 100%; 27.3 to 36 min, 100 to 0%; 36 to 41 min, 0%. The coupled 6490 triple quadrupole LC/MS device was operated with the following optimized source parameters: gas temperature, 50°C; gas flow, 11 liters/min; nebulizer pressure, 20 lb/in^2^; sheath gas temperature, 300°C; capillary voltage, 1,800 V; nozzle voltage, 2,000 V; iFunnel RF voltage, 150/50 V; and fragmentor voltage, 380 V. Based on a synthetic standard, Q was detected at a retention time of 11.5 min and identified based on two transitions: precursor *m/z* 410, products *m/z* 163 and 295. Quantitation of Q was achieved by normalizing the area under the curve (AUC) for the signal intensity of Q against the total AUC for canonical ribonucleosides U, C, A, and G for each injected sample. Analysis of each sample condition was performed with 12 biological replicates, and data are presented as means and standard errors (SEMs).

### Bisulfite sequencing of ${\text{tRNA}}_{\text{GUC}}^{\text{Asp}}$.

Total RNA isolation and bisulfite conversions were performed using a previously described protocol (19). Bisulfite-treated tRNAs were reverse transcribed by using a tRNA 3′-specific stem-loop primer and amplified with primers that bind only to the deaminated sequences at the 5′ end (universal primer and a specific ${\text{tRNA}}_{\text{GUC}}^{\text{Asp}}$ stem-loop primer). Amplicons (20) under each condition were subcloned into a pGEM-T Easy (Promega) vector and sequenced (Multi-Disciplinary Laboratories Unit, Bruce Rappaport Faculty of Medicine, Technion).

### APB Northern blotting to detect ${\text{tRNA}}_{\text{GUG}}^{\text{His}}$.

Acryloyl aminophenylboronic acid gels were prepared and run with a few modifications according to Igloi and Kossel (99). Briefly, 15 μg of RNA was deacetylated in 100 mM Tris-HCl (pH 9) for 30 min at 37°C. RNA was ethanol precipitated and resuspended in 10 μl DEPC-treated water and 1× RNA loading dye (Fermentas). Samples were then denatured for 10 min at 70°C and run at 4°C on Tris-acetate EDTA (TAE) buffer, 8 M urea, 15% acrylamide, and 5 mg/ml aminophenylboronic acid (Sigma) on Bio-Rad mini gels. The gel was run at 4°C at 75 V for 7 h until the bromophenol blue reached the bottom of the gel. The gels were then stained with ethidium bromide in 1× TAE buffer for 20 min and then visualized for equal loading of samples. The gels were destained with ultrapure water for 20 min, and samples were transferred to a Hydrobond-XL membrane (GE health care) by electrotransfer using 0.5× TAE as the transfer buffer for 45 min at 150 V. The membrane was cross-linked by UV using 120 mJ (Stratagene UV linker) and hybridized twice for 15 min each in 5 ml hybridization buffer (20 mM sodium phosphate buffer [pH 7.3], 300 mM NaCl, 1% SDS), followed by the addition of 150 μg/ml heat-denatured salmon sperm DNA (ssDNA) to the hybridization buffer and blocking for 1 h at 60°C. The membrane was then incubated with 15 pmol of biotinylated tRNA probes prepared against ${\text{tRNA}}_{\text{GUG}}^{\text{His}}$ and incubated at 60°C for 16 h. The membrane was then washed for 10 min with 5 ml wash buffer (20 mM sodium phosphate buffer [pH 7.3], 300 mM NaCl, 2 mM EDTA, 0.5% SDS) at 60°C and then incubated in hybridization buffer once at RT for 10 min. The membrane was then incubated in streptavidin-horseradish peroxidase (HRP) conjugate in 5 ml hybridization buffer (1:5,000) for 30 min followed by 2 washes for 10 min. The membranes were incubated with enhanced chemiluminescence reagent (Bio-Rad) and then covered in plastic wrap with the RNA side facing upwards; the blots were exposed to X-ray films and developed using a film processor.

### Acid denaturing gel electrophoresis for ${\text{tRNA}}_{\text{GUC}}^{\text{Asp}}$.

Acidic gels were prepared and run according to a modified protocol (100). Briefly, 15 μg of RNA was deacetylated in 100 mM Tris-HCl [pH 9] for 30 min at 37°C. Tubes were briefly centrifuged, and 5 μl of 2× acidic loading dye (8 M urea, 0.1 M sodium acetate [pH 4.8], 0.05% bromophenol blue, and 0.05% xylene cyanol) were added to each sample in the tube. The gel was run at 75 V for 4 to 5 h at 4°C with acid-TAE running buffer. The gels were blotted and hybridized according to the protocol described above with biotinylated probes for ${\text{tRNA}}_{\text{GUC}}^{\text{Asp}}$.

### Modeling of EhQTRT1 and EhQTRTD1.

Swiss Model & Phyre servers were used for template assessment and modeling of the homodimeric interface of the protein structures. The structure of EhQTRT1 was modeled using a combination of homology and *ab initio* model building approaches. The structure was modeled with high confidence using the high-resolution crystal structure of queuine tRNA-ribosyltransferase (EC 2.4.2.292) (tRNA-guanine [tm1561]) from Thermotoga maritima (PDB 2ASH). The structure of EhQTRTD1 was modeled using the crystal structure of QTRT2, the noncatalytic subunit of murine tRNA-guanine transglycosylase (PDB 6FV5).

### Modeling of the EhTGT complex.

The homodimer models of both the proteins EhQTRT1 and EhQTRTD1 were energy minimized using AMBER 14 molecular dynamics software (https://ambermd.org/index.php). Using the lowest energy structures, we performed the protein-protein docking experiment using the cluspro server (https://cluspro.org/login.php). We checked the docking for the all the possible conformations and defining the active site. The cluspro algorithm considers greedy clustering of ligand positions after obtaining the best poses by rotating the ligand using a grid. We have used the best poses from the top two clusters. The images were created using PyMOL software (https://pymol.org/2/).

### Data availability.

RNA-Seq data have been deposited at the Gene Expression Omnibus under accession number GSE142211. Supplemental data are available at DRYAD (https://datadryad.org/stash/share/Y6qAAIhEzUcPNlK16zSmOvPJyjd0bqUcPUz3mNw1fCg).
